# Thermal synthesis of conversion-type bismuth fluoride cathodes for high-energy-density Li-ion batteries

**DOI:** 10.1038/s42004-021-00622-y

**Published:** 2022-01-11

**Authors:** Julian F. Baumgärtner, Frank Krumeich, Michael Wörle, Kostiantyn V. Kravchyk, Maksym V. Kovalenko

**Affiliations:** 1 Laboratory of Inorganic Chemistry, Department of Chemistry and Applied Biosciences, ETH Zürich, Vladimir-Prelog-Weg 1, CH-8093 Zürich, Switzerland; 2 Laboratory for Thin Films and Photovoltaics, Empa – Swiss Federal Laboratories for Materials Science and Technology, Überlandstrasse 129, CH-8600 Dübendorf, Switzerland

**Keywords:** Solid-phase synthesis, Batteries, Batteries

## Abstract

Towards enhancement of the energy density of Li-ion batteries, BiF_3_ has recently attracted considerable attention as a compelling conversion-type cathode material due to its high theoretical capacity of 302 mAh g^−1^, average discharge voltage of *ca*. 3.0 V *vs*. Li^+^/Li, the low theoretical volume change of *ca*. 1.7% upon lithiation, and an intrinsically high oxidative stability. Here we report a facile and scalable synthesis of phase-pure and highly crystalline orthorhombic BiF_3_ *via* thermal decomposition of bismuth(III) trifluoroacetate at T = 300 °C under inert atmosphere. The electrochemical measurements of BiF_3_ in both carbonate (LiPF_6_-EC/DMC)- and ionic liquid-based (LiFSI-Pyr_1,4_TFSI) Li-ion electrolytes demonstrated that ionic liquids improve the cyclic stability of BiF_3_. In particular, BiF_3_ in 4.3 M LiFSI-Pyr_1,4_TFSI shows a high initial capacity of 208 mA g^−1^ and capacity retention of *ca*. 50% over at least 80 cycles at a current density of 30 mA g^−1^.

## Abstract (web summary)

Bismuth fluoride is a promising cathode material for lithium ion batteries due to its high theoretical capacity and cycling stability, but low-cost production methods are needed for potential commercialization. Here, the authors report a synthetic method to grow pure BiF_3_ via thermal decomposition of bismuth trifluoroacetate.

## Introduction

At present, the replacement of intercalation-type cathodes by their conversion-type counterparts is pursued as a path for improving the energy density of Li-ion batteries (LiBs)^1–5^. Among the plethora of conversion-type cathodes investigated, metal fluorides (Fe^6–8^, Cu^9–11^, Bi^12–14^, Ni^15^) have come into major research spotlight as they possess compelling properties, such as intrinsically high oxidative stability, high lithiation potentials of > 2 V vs. Li^+^/Li as compared to the voltages of transition metal oxides or sulfides, and high theoretical capacities in the range of 302–712 mAh g^–1 ^^2,3,16–18^. Consequently, in combination with a metallic lithium anode, fluorides may offer energy densities of up to 890–1680 Wh kg^−1^, which is significantly higher than that of intercalation-type cathodes such as LiFePO_4_ (578 Wh kg^−1^), LiNi_1/3_Mn_1/3_Co_1/3_O_2_ (610 Wh kg^−1^), and LiCoO_2_ (546 Wh kg^−1^). Overall, although the reversibility of conversion reactions of metal fluorides has been demonstrated, most of them possess poor capacity retention and limited cycle life, which is primarily caused by large volume changes and associated deterioration of the mechanical contact between the active material and conductive additive upon lithiation/delithiation.

**Fig. 1:**
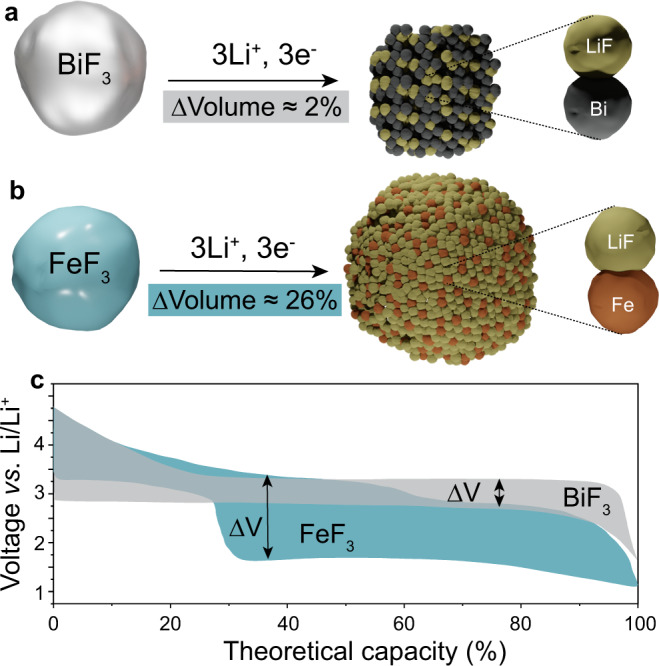
Comparison of BiF_3_ and FeF_3_ conversion-type cathodes for Li-ion batteries. **a**, **b** Schematics of the volume expansion of BiF_3_ (**a**) and FeF_3_ (**b**) upon their lithiation. **c** The typical voltage profiles (taken from Ref. ^24,25^.) and associated voltage hysteresis of BiF_3_ and FeF_3_ cathodes upon discharge (lithiation) and charge (delithiation).

BiF_3_ stands out from a range of conversion-type fluorides cathode materials because of its high theoretical capacity of 302 mAh g^−1^, owing to Bi^3+^/Bi^0^ redox process at 3.0 V *vs*. Li^+^/Li and low theoretical volume change of ca. 1.7% upon lithiation (Fig. 1a)^2,19–21^. The volume change upon delithiation is comparable to conventional intercalation-type cathodes such as LiFePO_4_ (*ca*. −6.8%)^22^, LiNi_0.5_Mn_0.3_Co_0.2_O_2_ (*ca*. −2.2%)^23^, and LiCoO_2_ (*ca*. 1.9%)^23^. For comparison, extensively studied FeF_3_ has much higher volume changes of up to 26% for three-electron operation (Fig. 1b)^2^. Additionally, BiF_3_ cathodes are characterized by low voltage hysteresis of ca. 0.4 V^24^, contrary to 1.5–2 V for FeF_3_ (Fig. 1c)^25–29^.

This study was motivated by (i) a lack of low-cost and robust synthesis methods of BiF_3_ and (ii) its well-recognized potential as a high-energy-density cathode material in Li-ion batteries. Traditionally, BiF_3_ is synthesized from various Bi^3+^ precursors such as Bi_2_O_3_ or BiOCl *via* treatment with concentrated HF^20,30,31^. Alternative solution-based approaches employ Bi(NO_3_)_3_ or Bi(NO_3_)(OH)_2_ and NH_4_F^32–34^. However, both HF and NH_4_F fluorine sources are hazardous and highly toxic and the employment of aqueous media often results in the formation of bismuth oxofluorides impurities through a hydrolysis reaction. Harnessing the storage potential of BiF_3_, at present, it is hindered by its poor capacity retention in carbonate-based Li-ion electrolytes, which is associated with the formation of Li_2_CO_3_ containing cathode-electrolyte interfaces (CEIs) upon discharge, catalyzed by metallic Bi^0,^^12,24,35^. In the context of minimization of these side reactions, herein, we sought to thoroughly test room-temperature ionic liquids (ILs) as alternative Li-ion electrolytes. Recently, Pasta *et al*^36^. demonstrated superior cycling stability for a similar conversion FeF_2_ cathode, caused by the formation of stable solid electrolyte interphase. Moreover, ILs have been found to provide exceptional long cycling stability for conventional intercalation-type electrodes^37–39^. However, to our knowledge, the electrochemical performance of BiF_3_ cathode in ILs has not yet been studied.

In this work, we present a novel facile and scalable synthesis synthetic route of BiF_3_ by the thermal decomposition of non-toxic, safe-to-handle, single-source molecular Bi trifluoroacetate (TFA) precursor under N_2_ atmosphere, yielding highly crystalline orthorhombic BiF_3_. The electrochemical performance of BiF_3_ was thoroughly assessed in both carbonate and IL Li-ion electrolytes. We determined that the BiF_3_ exhibits high cyclic stability as cathode material in lithium-ion batteries based on 4.3 M LiFSI-Pyr1, 4TFSI IL, delivering a high initial capacity of 208 mAh g^–1^. Half of this capacity was retained after prolonged operation over 80 cycles at a current density of 30 mA g^−1^.

## Results and discussion

### Synthesis and characterization of BiF_3_

**Fig. 2:**
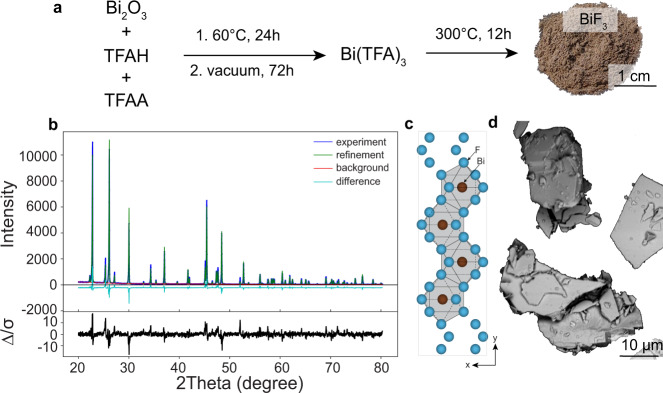
Synthesis and characterization of BiF_3_. **a** Schematic of the BiF_3_ synthesis (trifluoroacetic acid and trifluoroacetic anhydride are named as TFAA and TFAH, respectively); **b** A comparison of the experimental (blue) and calculated (green) powder diffraction pattern of BiF_3_ together with the difference (cyan) and the background (red), as obtained from the Rietveld refinement with GSAS-II^43^. **c** Orthorhombic structure of BiF_3_. **d** SEM image of the BiF_3_ particles.

Fig. 2a outlines the synthesis of BiF_3_ *via* thermal decomposition of Bi(TFA)_3_ as a single-source precursor under N_2_ atmosphere (12 h at 300 °C). Bi(TFA)_3_ was synthesized according to the procedure given in Ref. ^40,41^. (see Methods Section and Supplementary Fig. 1 for details). Powder X-ray diffraction (XRD) measurements along with Rietveld refinement^42^ confirmed the formation of chemically pure, highly crystalline orthorhombic modification of BiF_3_ (*Pnma*, space group no. 62, *a* = 6.5604 Å *b* = 7.0174 Å, *c* = 4.8450 Å, ICSD No. 1269, Fig. 2b, c). The R(F) factor is 7% (see Methods section for details). No significant broadening of the reflections associated with crystallite size effects is detected. The powder pattern shows a strong preferred orientation of the crystallites along the [010]-direction, which is in line with the flake morphology of BiF_3_ powder (mean particle size is 4.9 μm), as shown in Fig. 2d. The BiF_3_ product is air-stable and only shows minor hydrolysis towards the formation of cubic BiO_x_F_3-2x_ phase even after several months on air (see Supplementary Fig. 2). In contrast, it was found that if the synthesis is carried out under air or if the precursor is ground under air, the synthesis yields different BiO_x_F_3-2x_ phases (Supplementary Fig. 3) with 0.4 < x < 0.6 (cubic, *Fm-*3*m*, space group no. 225) and x = 1 (tetragonal, P4/nmm, space group no. 129) along with the formation of a white, amorphous sublimate on the quartz tube outside the oven (Supplementary Fig. 4).

**Fig. 3:**
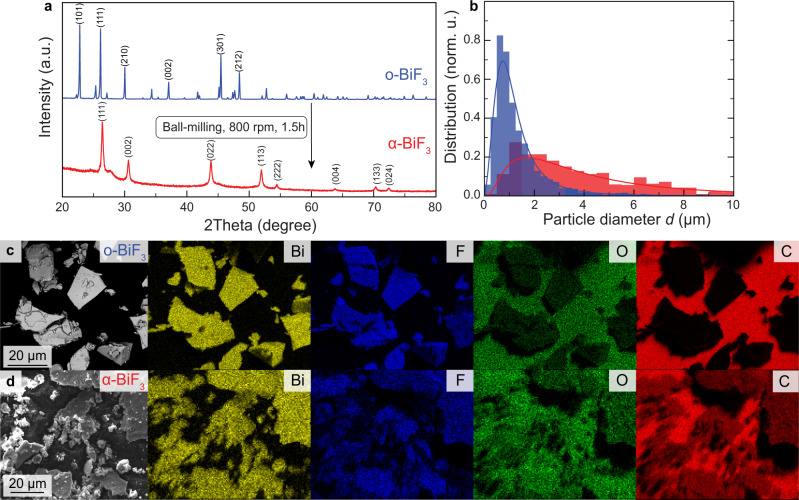
Characterization of BiF_3_ before and after ball milling. **a** Powder XRD patterns, (**b**) the particle size distributions (see Supplementary Figs. 11, 12 and Supplementary Tables 1, 2 for fitting details), and (**c**, **d**) SEM/EDX images of BiF_3_ before (**c**) and after ball-milling (**d**).

Since BiF_3_ is an electrically insulating compound, the synthesized μm-sized BiF_3_ particles were reasoned to be too large for their employment in electrodes. Consequently, to reduce the particle size and establish sufficient contact with the conductive additives, o-BiF_3_ was dry ball-milled with multi-walled carbon nanotubes (CNTs) and carbon black (CB) at 800 rpm for 1.5 h under Ar atmosphere. Interestingly, ball-milling causes the transformation of the orthorhombic structure of BiF_3_ into the cubic one (*Fm-*3*m*, space group no. 225, Fig. 3a). These results are in contrast with previous findings by Bervas et al^19^., who reported the formation of the high-pressure tysonite modification ($P\bar 3{{{{{{{\mathrm{c1}}}}}}}}$, space group no. 165)^44,45^ after high-energy ball-milling at 1100 rpm. Obviously, considering that we conducted ball-milling at a lower speed of 800 rpm, the generated pressure was insufficient to transform o-BiF_3_ into the high-pressure modification. Due to the fact that XRD patterns of ball-milled BiF_3_ can be assigned to either α-BiF_3_^41^, or BiO_x_F_3-2x_ with 0.4 < x < 0.6^12^, two phases cannot be distinguished based on powder XRD analysis because both lattice parameters of the reference compounds and the reflection intensities in the XRD patterns are almost identical. However, EDX measurements indicated that the O content in cubic BiF_3_ is relatively low (see Fig. 3c, d and Supplementary Fig. 5–9), suggesting that ball-milling yields the formation of α-BiF_3_ phase with minor O impurities rather than a BiO_x_F_3-2x_ phase. Slightly enhanced amounts of oxygen in cubic BiF_3_ as compared to orthorhombic BiF_3_ probably originate from the presence of adsorbed water on the surface of CNTs and CB conducting additives used for ball-milling. Notably, ball-milling of BiF_3_ induced significant broadening of XRD reflections, indicating the reduction of crystallite sizes and the presence of considerable strain within the material (Fig. 3a). As the product is not monodisperse, a meaningful quantification of strain and crystallite size was not possible. Importantly, SEM measurements revealed that the mean particle size of BiF_3_ after ball-milling decreases from 4.9 μm to 1.2 μm and the distribution becomes much narrower from 4.9 to 1.0 μm (Fig. 3b). Also, ball-milling caused the formation of cracks in the BiF_3_ particles (see Supplementary Fig. 7 and 8), thus allowing conductive additives to intermix well with the BiF_3_ active material (Fig. 3c). Carbon EDX mapping after ball-milling clearly shows that carbon coats the entire surface of BiF_3_ particles. Additionally, to further investigate the contact between the active material and conductive additive on a nanoscale level, TEM measurements of BiF_3_ after ball-milling were performed. Supplementary Fig. 10 shows that the obtained BiF_3_/C powder consist of a significant amount of BiF_3_ nanoparticles (*ca*. 20 nm), which are embedded into a CNT and CB matrix.

### Electrochemical performance

For the electrochemical measurements, BiF_3_ electrodes were prepared by ball-milling of BiF_3_/C powder with (polyvinylidene difluoride) pVdF binder in (N-Methylpyrrolidone) NMP solvent, and the resulting slurries were cast onto an Al foil current collector via paint brushing, followed by drying at 80 °C for 12 h under vacuum. Coin-type cells were employed for the electrochemical measurements. The cell consisted of a lithium disk as a counter and reference electrode and a BiF_3_ working electrode. A glass-fiber separator was placed in between the two electrodes and impregnated with Li-ion electrolyte. All the electrochemical tests were performed in the voltage range of 2 – 4 V *vs*. Li^+^/Li at a current density of 30 mA g^−1^.

**Fig. 4:**
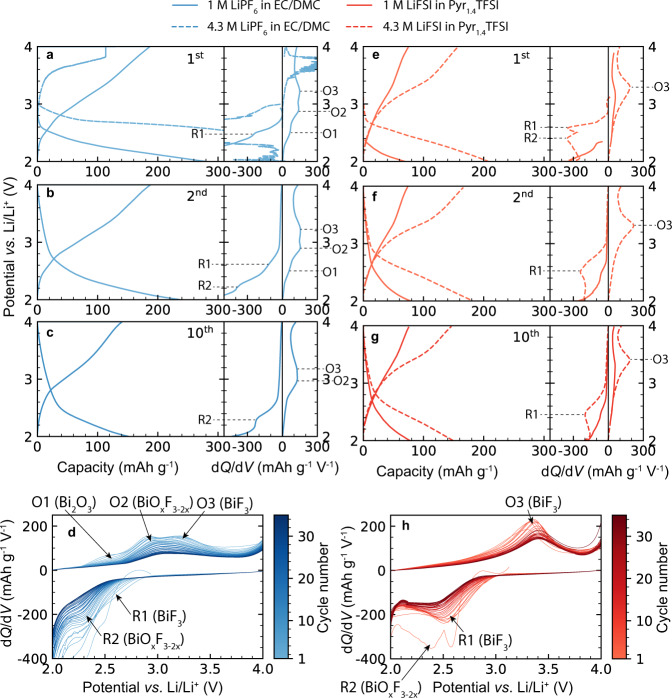
Electrochemical performance. Charge and discharge voltage profiles and associated *dQ/dV* curves of BiF_3_ cathode measured in LiPF_6_ (1 M and 4.3 M)-EC/DMC and LiFSI (1 M and 4.3 M)-Pyr_1,4_TFSI Li-ion electrolytes for the first (**a**, **e**), second (**b**, **f**) and tenth (**c**, **g**) cycles. Combined d*Q*/d*V* plots measured upon cycling of BiF_3_ cathode in 1 M LiPF_6_-EC/DMC Li-ion electrolyte for first the 35 cycles; (**d**) and 4.3 M LiFSI (**h**) in Pyr_1,4_TFSI Li-ion electrolytes. The BiF_3_/Li half cells were cycled at a current density of 30 mA g^−1^ in the voltage range of 2‒4 V vs. Li^+^/Li. The electrochemical performance of BiF_3_ measured in other carbonate-based electrolytes (1 M LiPF_6_ in EC/DMC + 3 wt-% FEC, EMC and EMC + 3 wt-% FEC) is given in Supplementary Fig. S14 for comparison.

Firstly, we assessed the electrochemical performance of BiF_3_ employing a conventional electrolyte (1 M LiPF_6_-EC/DMC). The charge and discharge voltage profiles, as well as corresponding differential capacity plots (d*Q*/d*V*) are summarized in Fig. 4 and rescaled in Supplementary Fig. 13. During the first discharge, two reduction peaks at 2.5 V (R1) and 2.2 V (R2) *vs*. Li^+^/Li were observed, which can be attributed to the reduction of BiF_3_^14,46^ and BiO_x_F_3-2x_^46^. The voltage difference between the two peaks of only 0.3 V indicates that the O content in BiO_x_F_3–2x_ must be rather low. The O impurities might originate for two reasons: the presence of minor quantities of amorphous BiO_x_F_3–2x_ in as-prepared BiF_3_/C powder or/and the fact that residual water from the electrolyte could also hydrolyze BiF_3_ to form BiO_x_F_3–2x_. The differential capacity then keeps decreasing to −750 mAh g^–^^1^ V^–^^1^ at 2 V *vs*. Li^+^/Li (Supplementary Fig. 13). This is presumably associated with the reduction of the carbonate electrolyte catalyzed by metallic Bi^0^. Of note, carbonate electrolytes, in particular cyclic carbonates, form Li_2_CO_3_ containing cathode-electrolyte interfaces (CEIs) upon reduction at 1.6–2 V in the presence of Bi^0^^38,52,53^. The capacity for the first cycle is 280 mAh g^–^^1^, which is close to the theoretical maximum of 302 mAh g^−1^ for BiF_3_.

Upon charge, several distinct electrochemical processes occur as follows from the appearance of the oxidation peaks at 2.5 V (O1), 2.8 V (O2) and 3.2 V (O3) *vs*. Li^+^/Li. The first small oxidation O1 peak at 2.5 V *vs*. Li^+^/Li can be associated with the oxidation of Bi^0^ to Bi_2_O_3_ in the presence of Li_2_CO_3_ $\left( {2Bi^0{{{{{{{\mathrm{ + }}}}}}}}3{{{{{{{\mathrm{Li}}}}}}}}_2CO_3\left( {{{{{{{{\mathrm{CEI}}}}}}}}} \right) \to {{{{{{{\mathrm{Bi}}}}}}}}_2O_3{{{{{{{\mathrm{ + }}}}}}}}3CO_2{{{{{{{\mathrm{ + }}}}}}}}6Li^ + {{{{{{{\mathrm{ + }}}}}}}}6e^ - } \right)$^35^. Next, the O2 peak at 2.8 V *vs*. Li^+^/Li is caused by the oxidation of Bi forming BiO_x_F_3-2x_ while the O3 peak at 3.2 V vs. Li^+^/Li is assigned to BiF_3_ formation^19,21,46^. A similar behavior with three distinct oxidation peaks has already been reported for BiO_x_F_3-2x_ cathodes^46^. At voltages above 3.9 V vs. Li^+^/Li, the differential capacity increases slightly again, pointing to electrolyte oxidation. Interestingly, the three oxidation peaks are only present in the first cycles. From the tenth cycle onwards, they merge into two peaks with a concomitant decrease of the discharge capacity down to 152 mAh g^–^^1^. Thus, the O1 peak (Bi^0^/Bi_2_O_3_) shifts to higher voltages and then disappears completely, while the O2 peak (Bi^0^/BiO_x_F3_-2x_) also shifts to higher voltages and prevails. The O3 peak (Bi^0^/BiF_3_) at 3.2 V vs. Li^+^/Li moves to slightly lower voltages of 3.0‒3.1 V vs. Li^+^/Li after 15 cycles and then shifts again to a higher voltage of 3.2–3.3 V vs. Li^+^/Li onwards. The shift in two directions is possibly caused by two independent phenomena. On the one hand, a shift to higher voltages upon cycling might be associated with loss of electrical contact of BiF_3_ part due to conversion reaction, eventually increasing the applied current density per electrochemically active sites of BiF_3_, thus resulting in higher overpotentials. On the other hand, the shift of O3 peak toward lower voltages, followed by its disappearance in the following cycling, can be explained by homogenization of Bi_2_O_3_ and BiF_3_ domains towards the formation of a BiO_x_F_3-2x_ phase $( {xBi_2O_3 + (3 - 2x)BiF_3\mathop{\longrightarrow} \limits^{Cycling} 3BiO_xF_{3 - 2x}} )$.

Next, we measured BiF_3_ employing IL Li-ion electrolyte based on lithium bis(fluorosulfonyl)imide (LiFSI) in 1-butyl-1-methylpyrrolidinium bis(trifluoromethylsulfonyl)imide (Pyr_1,4_TFSI). As it was previously reported, imidazolium^47–49^, and pyrrolidinium (Pyr)^50–53^ IL electrolytes have the advantage of not forming oxygen-containing decomposition products that might help to overcome the partial formation of BiO_x_F_3-2x_ phases. Contrary to the results obtained with carbonate-based electrolyte, the employment of the IL counterpart resulted in only one reduction peak at 2.4 V (R1) upon initial discharge. Further decrease of the differential capacity at *ca*. 2 V (Fig. 4e, h) might be associated with the formation of CEI or another side reaction. Yet, the extent of this side reaction is far less pronounced as compared to measurements performed in LiPF_6_ in EC/DMC electrolyte. Interestingly, upon the subsequent charge, no O1 oxidation peak at 2.5 V vs. Li^+^/Li (Bi^0^/Bi_2_O_3_) was observed. Only one broad oxidation peak was measured at 3.2 V vs. Li^+^/Li (O3), which is associated with the oxidation of Bi^0^ to BiF_3_. This observation indicates that the employment of oxygen-free IL electrolyte hinders the formation of Li_2_CO_3_ and, therefore, its chemical reaction with Bi, yielding Bi_2_O_3_.

Next, we assessed the impact of the salt concentration in both carbonate and IL electrolytes on the electrochemical performance of BiF_3_ cathode. Cells were prepared with 4.3 M LiPF_6_ in EC/DMC + 3 wt-% FEC and 4.3 M LiFSI in Pyr_1,4_TFSI electrolytes, accordingly. For effective penetration of the cathode by the highly viscous electrolytes, the prepared coin-type cells were heat-treated at 75 °C for 24 h prior to measurements.

The initial discharge of BiF_3_ in 4.3 M carbonate electrolyte resulted in a pronounced reduction plateau at 2.6–2.7 V *vs*. Li^+^/Li, corresponding to an initial discharge capacity of 481 mAh g^–^^1^ (Fig. 4a). This is far higher than the theoretical discharge capacity of 302 mAh g^−1^. Upon subsequent charge, a pronounced oxidation peak at about 3.7 V vs. Li^+^/Li was observed, after which the cell short-circuited at a capacity of 114 mAh g^–^^1^, caused presumably by Li dendrite formation. In contrast to carbonate media, BiF_3_ cathodes in highly concentrated IL electrolytes were characterized by stable electrochemical performance. Galvanostatic discharge of BiF_3_ in 4.3 M LiFSI in Pyr_1,4_TFSI revealed the formation of two small reduction peaks at 2.6 V (R1) and 2.4 V (R2) *vs*. Li^+^/Li, which can be assigned to the reduction of BiF_3_ and BiO_x_F_3-2x_, respectively. The differential capacity curve does not drop below –300 mAh g^–^^1^ V^–^^1^, indicating that no significant amount of CEI is formed. The total capacity of BiF_3_ cathode after initial discharge was *ca*. 208 mAh g^-1^. Upon charge, only one oxidation peak O1 at 3.3 V *vs*. Li^+^/Li was observed, associated with oxidation of Bi^0^ to BiF_3_. In subsequent cycles, the reduction peak R1 shifts slightly to 2.5 V vs. Li^+^/Li while oxidation peak O1 shifts to 3.4 V vs. Li^+^/Li, resulting in a slight increase of voltage hysteresis from 0.8 V to 0.9 V. At voltages above 3.9 V *vs*. Li^+^/Li, the differential capacity increases slightly again, which is probably associated with electrolyte decomposition. Overall, the differential capacity plots measured for BiF_3_ in the highly concentrated IL electrolyte do not show any noticeable formation of CEIs upon both reduction and oxidation.

**Fig. 5:**
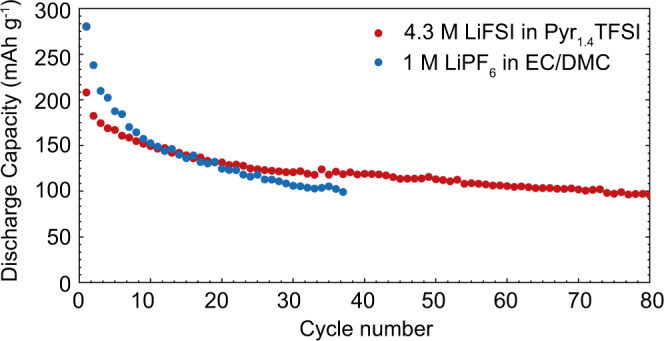
Cycling stability measurements. Cycling stability of BiF_3_ cathode measured in 1 M LiPF_6_-EC/DMC and 4.3 M LiFSI-Pyr1,4TFSI electrolytes at a current density of 30 mA g^‒1^ within the voltage range of (2‒4 V *vs*. Li^+^/Li). Drop of charge-storage capacity below 100 mAh g^−1^ was considered as a cutoff criterion.

We then compared the cycling stabilities of BiF_3_ employing 1 M LiPF_6_-EC/DMC and 4.3 M LiFSI-Pyr_1,4_TFSI electrolytes. As follows from Fig. 5, BiF_3_ with carbonate-based electrolyte exhibited a high initial discharge capacity of 280 mAh g^‒1^. However, this capacity fades rapidly below 100 mAh g^–^^1^ after 38 cycles, corresponding to the capacity retention of 36%. On the contrary, BiF_3_ measured in IL electrolytes displayed significantly higher capacity retention of 46% over 80 cycles, although its initial capacity is slightly lower (208 mAh g^–^^1^). Comparison of the electrochemical performance of BiF_3_ with reported systems comprising BiF_3_^12,14,19,21,24^ iron fluorides^7,17,54^ or alkali iron fluorides^55,56^ can be found in the supporting information (see Supplementary Table 3). Notably, superior cycling stability of BiF_3_ was revealed in 1 M LiFSI-Pyr_1,4_TFSI electrolyte over 180 cycles, with the capacity ranging between 80 and 90 mAh g^-1^ (see Supplementary Fig. 15). The enhanced electrochemical performance of BiF_3_ in LiFSI-Pyr_1,4_TFSI electrolytes can be attributed to the formation of a stable CEI that effectively suppresses continuous electrolyte reduction during battery cycling, as observed for carbonate-based electrolytes.

## Conclusions

In summary, we have reported a facile, low-cost, and scalable synthesis of crystalline o-BiF_3_ *via* thermal decomposition of a single-source Bi(TFA)_3_ precursor under N_2_ atmosphere. When the synthesis is performed in air, the formation of BiO_x_F_3-2x_ with different amounts of oxygen (0.4 < x < 0.6) takes place. Side by side comparison of the electrochemical performance of BiF_3_ in carbonate- and IL-based electrolytes revealed that the use of ILs significantly improves cyclic stability of BiF_3_. In particular, a high initial capacity of *ca*. 208 mAh g^−1^ was obtained at a current density of 30 mA g^–1^ (∼C/10), and half of this capacity was retained after 80 cycles. We speculate that employment of IL overcomes the major constraint of carbonate-based electrolytes associated with accumulation of Li_2_CO_3_ CEIs at 1.6–2 V vs. Li^+^/Li, which hinders Li-ion percolation from the electrolytes towards the surface of BiF_3_ particles.

## Methods

### Synthesis of Bi(TFA)_3_

The synthesis was carried out as reported elsewhere^40^, with slight modifications^14^. Bi_2_O_3_ (10.0 g, 21.5 mmol) was mixed with trifluoroacetic acid (10 ml, 139 mmol) and trifluoroacetic anhydride (20 ml, 139 mmol) in a three-neck flask, connected to a Schlenk line, and stirred under N_2_ atmosphere at 60 °C for 24 h. The oxide fully dissolved, forming a viscous, brownish solution. The solvents were evaporated under vacuum, resulting in a white powder (yield: 96%).

### Synthesis of BiF_3_

The as-synthesized Bi(TFA)_3_ (3.30 g, 6.03 mmol) was added inside an N_2_-filled glovebox (O_2_, H_2_O < 0.1 ppm) to an alumina crucible and placed into a silica quartz tube that was fused on one side and open on the other. The open end was then sealed with a valve and transferred into a tube furnace. The valve was used to release the gaseous decomposition products. The sample was heated under N_2_ flow from RT to 100 °C at 300 °C h^−1^ at 300 °C h^−1^ where it was kept for 2 h, and then heated at 50 °C h^−1^ to 300 °C where it was kept for 12 h before cooling to RT at 300 °C h^−1^, yielding a brown powder (1.60 g, 6.00 mmol, 100%). The yield was determined by weighing the product before and after the heat treatment. The stability of BiF_3_ was tested under air by storing the product in a 5 ml glass vial under air for 3 months.

### Electrochemical measurements

In a typical cathode preparation, BiF_3_ (37.5 mg, 50 wt%) and the conductive additives carbon black (CB, 15 mg, 20 wt%) and multi-walled carbon nanotubes (CNT, 50–90 nm, 15 mg, 20 wt%) were dry-mixed under Ar in a planetary ball mill (Fritsch, Pulverisette 7) in an anatase beaker with 28 anatase balls for 1:30 h at 800 rpm. Carbon nanotubes were used to enhance electron percolation in the BiF_3_ cathode. Afterwards, polyvinylidene fluorine (pVdF, 7.5 mg, 10 wt-%) was added as a binder. N-methyl-2-pyrrolidone (NMP, 650 μL, 900 wt-%) were added as a solvent and ball-milled under Ar for 1:30 h at 500 rpm to form a slurry. This slurry was then applied by brushing it onto carbon-coated Al foil (12 mm Ø). The painted current collectors were dried under air at 75 °C for 1 h and then dried under vacuum at 80 °C for 18 h. The final loading of BiF_3_ was ca. 0.1 mg cm ^−^^2^. In an Ar-filled glovebox, the cathode was incorporated into an air-tight coin-type cell. Elemental Li coins (12 mm Ø) were used as counter and reference electrodes. Typically, 200–300 μL of ion-conducting electrolyte was added onto the glass microfiber (Whatman) separator. IL-containing cells were then heated under Ar for 24 h at 75 °C to ensure good wetting of the cell. Likewise, the 4.3 M LiPF_6_ in EC/DMC was heated under Ar for 20 min at 75 °C to ensure good wetting. If the cell was heated longer, gas evolution caused the cell to burst. Moreover, every cell was prepared at least twice to ensure the reproducibility of the results. Galvanostatic cycling at 30 mA g^−1^ was performed in a voltage range between 2 and 4 V on a multichannel potentiostat/ galvanostat from Biologic (MPG2).

### Transmission and Scanning electron microscopy (TEM and SEM) measurements

TEM measurements were performed on the Talos F200X (ThermoFisher Scientific, FEG, *U*_acc_ = 200 kV). SEM measurements of the as-obtained samples were done on a Quanta 200 F microscope (Thermo Fisher Scientific) operated at an acceleration voltage Vacc = 20 kV. Energy-dispersive X-ray spectroscopy (EDXS) was performed with an Octane SDD detector (EDAX, Ametec) attached to the microscope column. For spectra recording and quantification (ZAF correction), the software Gemini (EDAX) was used.

### Powder X-ray diffraction measurements

The powder XRD patterns were collected at RT on a Stoe STADI P powder X-ray diffractometer (Cu Kα_1_ radiation, λ = 1.540598 Å, focusing germanium monochromator) equipped with a Dectris Mythen 1 K silicon strip detector.

### Rietveld Refinement

The composition of the BiF_3_ product was analyzed based on the powder XRD pattern (Cu Kα1 radiation, λ = 1.540598 Å) by a Rietveld refinement with the GSAS II program^43^. For the refinement, the starting model of o-BiF_3_ given by Greis et. al^30^. was used (space group Pnma, a = 6.5614(4) Å, b = 7.0153(5) Å, c = 4.8414(3) Å, Bi on 4c with x = 0.3547(1), y = 0.25, z = 0.0349(1); F1 on 4c with x = 0.5361(17), y = 0.25, z = 0.6271(26) and F2 on 8d with x = 0.1652(14), y = 0.0577(11), z = 0.3528(15)). Except the lattice constants and the displacement parameters, none of the structural parameters was refined. The unit cell parameters obtained by the refinement were: a = 6.56037(8) Å, b = 7.01741(16) Å, c = 4.84501(7) Å. The displacement parameters of the F-atoms were constrained to be equal and to be 1.67 times larger than the one for the Bi-atom. The displacement parameters for Bi refined to U_iso_ = 0.0272(5) Å^2^ and accordingly the displacement parameters for the F-atoms are U_iso_ = 0.0453 A^2^. The Figures of Merit were: reduced χ2 = 4.46, wR = 0.14574, R(F) = 0.0744, R(F^2^) = 0.27704 (on 73 reflections), N_obs_ = 4020, N_vals_ = 21. The March-Dollase ratio for a preferred orientation along the [010]-direction is 1.543.

### Particle Size Distributions

ImageJ was used to count the individual particles. Because of the strongly anisotropic shape, every particle was measured with two lines perpendicular to each other to get an average size for the particle. The SEM images used for counting can be seen in Supplementary Figs 11, 12. The sample sizes were *N* = 678 for the distribution before ball-milling and *N* = 2231 after ball-milling. The fitting of the particle size distribution was carried out using the reliability module in python^57^. Parameters were estimated using a maximum likelihood estimation. The equations for the particle size distributions used for fitting are given as:

1 $${{{{{{{\mathrm{Lognormal}}}}}}}}\;{{{{{{{\mathrm{2}}}}}}}}\;{{{{{{{\mathrm{P}}}}}}}}\;f\left( t \right) = \frac{1}{{\sigma t\sqrt {2\pi } }}\exp \left[ { - \frac{1}{2}\left( {\frac{{\ln \left( t \right) - \mu }}{\sigma }} \right)^2} \right]$$

2 $${{{{{{{\mathrm{Lognormal}}}}}}}}\;{{{{{{{\mathrm{3}}}}}}}}\;{{{{{{{\mathrm{P}}}}}}}}\;f\left( t \right) = \frac{1}{{\sigma \left( {t - \gamma } \right)\sqrt {2\pi } }}\exp \left[ { - \frac{1}{2}\left( {\frac{{\ln \left( {t - \gamma } \right) - \mu }}{\sigma }} \right)^2} \right]$$

3 $${{{{{{{\mathrm{Loglogistic}}}}}}}}\;{{{{{{{\mathrm{3}}}}}}}}\;{{{{{{{\mathrm{P}}}}}}}}\;f\left( t \right) = \frac{{\left( {\frac{\beta }{\alpha }} \right)\Big( {\frac{{t - \gamma }}{\alpha }} \Big)^{\beta - 1}}}{{\left( {1 + \left( {\frac{{t - \gamma }}{\alpha }} \right)^\beta } \right)^2}}$$

4 $${{{{{{{\mathrm{Loglogistic}}}}}}}}\;2\;{{{{{{{\mathrm{P}}}}}}}}\;f\left( t \right) = \frac{{\left( {\frac{\beta }{\alpha }} \right)\Big( {\frac{t}{\alpha }} \Big)^{\beta - 1}}}{{\left( {1 + \left( {\frac{t}{\alpha }} \right)^\beta } \right)^2}}$$

5 $${{{{{{{\mathrm{Gamma}}}}}}}}\;{{{{{{{\mathrm{3}}}}}}}}\;{{{{{{{\mathrm{P}}}}}}}}\;f\left( t \right) = \frac{{\gamma t^{\beta - 1}}}{{{{{{{{{\mathrm{{\Gamma}}}}}}}}}\left( {\frac{\beta }{\gamma }} \right)\alpha ^\beta }}e^{ - \left( {\frac{t}{\alpha }} \right)^\gamma }\;{{{{{{{\mathrm{with}}}}}}}}\;{\Gamma}\left( {\frac{\beta }{\gamma }} \right) = \mathop {\smallint }\limits_0^\infty t^{\frac{\beta }{\gamma } - 1}e^{ - t}{{{{{{{\mathrm{d}}}}}}}}t$$

6 $${{{{{{{\mathrm{Weibull}}}}}}}}\;{{{{{{{\mathrm{3}}}}}}}}\;{{{{{{{\mathrm{P}}}}}}}}\;f\left( t \right) = \left( {\frac{\beta }{\alpha }} \right)\Bigg( {\frac{{t - \gamma }}{\alpha }} \Bigg)^{\beta - 1}e^{ - \left( {\frac{{t - \gamma }}{\alpha }} \right)^\beta }$$

7 $${{{{{{{\mathrm{Exponential}}}}}}}}\;{{{{{{{\mathrm{2}}}}}}}}\;{{{{{{{\mathrm{P}}}}}}}}\;f\left( t \right) = \lambda e^{ - \lambda \left( {t - \gamma } \right)}$$

8 $${{{{{{{\mathrm{Gamma}}}}}}}}\;{{{{{{{\mathrm{2}}}}}}}}\;{{{{{{{\mathrm{P}}}}}}}}\;f\left( t \right) = \frac{{t^{\beta - 1}}}{{{{{{{{{\mathrm{{\Gamma}}}}}}}}}\left( \beta \right)\alpha ^\beta }}e^{ - \frac{t}{\alpha }}\;{{{{{{{\mathrm{with}}}}}}}}\;{{{{{{{\mathrm{{\Gamma}}}}}}}}}\left( \beta \right) = \mathop {\smallint }\limits_0^\infty t^{\beta - 1}e^{ - t}{{{{{{{\mathrm{d}}}}}}}}t$$

9 $${{{{{{{\mathrm{Weibull}}}}}}}}\;{{{{{{{\mathrm{2}}}}}}}}\;{{{{{{{\mathrm{P}}}}}}}}\;f\left( t \right) = \left( {\frac{\beta }{\alpha }} \right)\bigg( {\frac{t}{\alpha }} \bigg)^{\beta - 1}e^{ - \left( {\frac{t}{\alpha }} \right)^\beta }$$

10 $${{{{{{{\mathrm{Exponential}}}}}}}}\;{{{{{{{\mathrm{1}}}}}}}}\;{{{{{{{\mathrm{P}}}}}}}}\;f\left( t \right) = \lambda e^{ - \lambda t}$$

11 $${{{{{{{\mathrm{Normal}}}}}}}}\;{{{{{{{\mathrm{2}}}}}}}}\;{{{{{{{\mathrm{P}}}}}}}}\;f\left( t \right) = \frac{1}{{\sigma \sqrt {2\pi } }}\exp \left[ { - \frac{1}{2}\left( {\frac{{t - \mu }}{\sigma }} \right)^2} \right]$$

12 $${{{{{{{\mathrm{Gumbel}}}}}}}}\;{{{{{{{\mathrm{2}}}}}}}}\;{{{{{{{\mathrm{P}}}}}}}}\;f\left( t \right) = \frac{1}{\sigma }e^{z - e^z}\;{{{{{{{\mathrm{with}}}}}}}}\;z = \frac{{t - \mu }}{\sigma }$$

## Supplementary information

Supplementary Information

## Data Availability

The data that support the plots within this paper and other finding of this study are available from the corresponding author upon reasonable request.
