# Supplementary material for: Thermal synthesis of conversion-type bismuth fluoride cathodes for high-energy-density Li-ion batteries
Source: Commun Chem. 2022 Jan 11;5:6. doi: 10.1038/s42004-021-00622-y (PMC9814757; doi:10.1038/s42004-021-00622-y)
Supplement: Supplementary file 1 — Supplementary Information [file 42004_2021_622_MOESM1_ESM.pdf]

*Supporting Information for*

# Thermal Synthesis of Conversion-Type Bismuth Fluoride Cathodes for High-Energy-Density Li-ion Batteries

*Julian F. Baumgärtner,[1,2] Frank Krumeich,[1] Michael Wörle,[1] Kostiantyn V. Kravchyk,[1,2] and Maksym V. Kovalenko[1,2*]*

[1] Laboratory of Inorganic Chemistry, Department of Chemistry and Applied Biosciences, ETH Zürich, Vladimir-Prelog-Weg 1, CH-8093 Zürich, Switzerland

[2] Laboratory for Thin Films and Photovoltaics, Empa – Swiss Federal Laboratories for Materials Science and Technology, Überlandstrasse 129, CH-8600 Dübendorf, Switzerland

**Corresponding Authors:**

*E-mails: mvkovalenko@ethz.ch and kravchyk@inorg.chem.ethz.ch

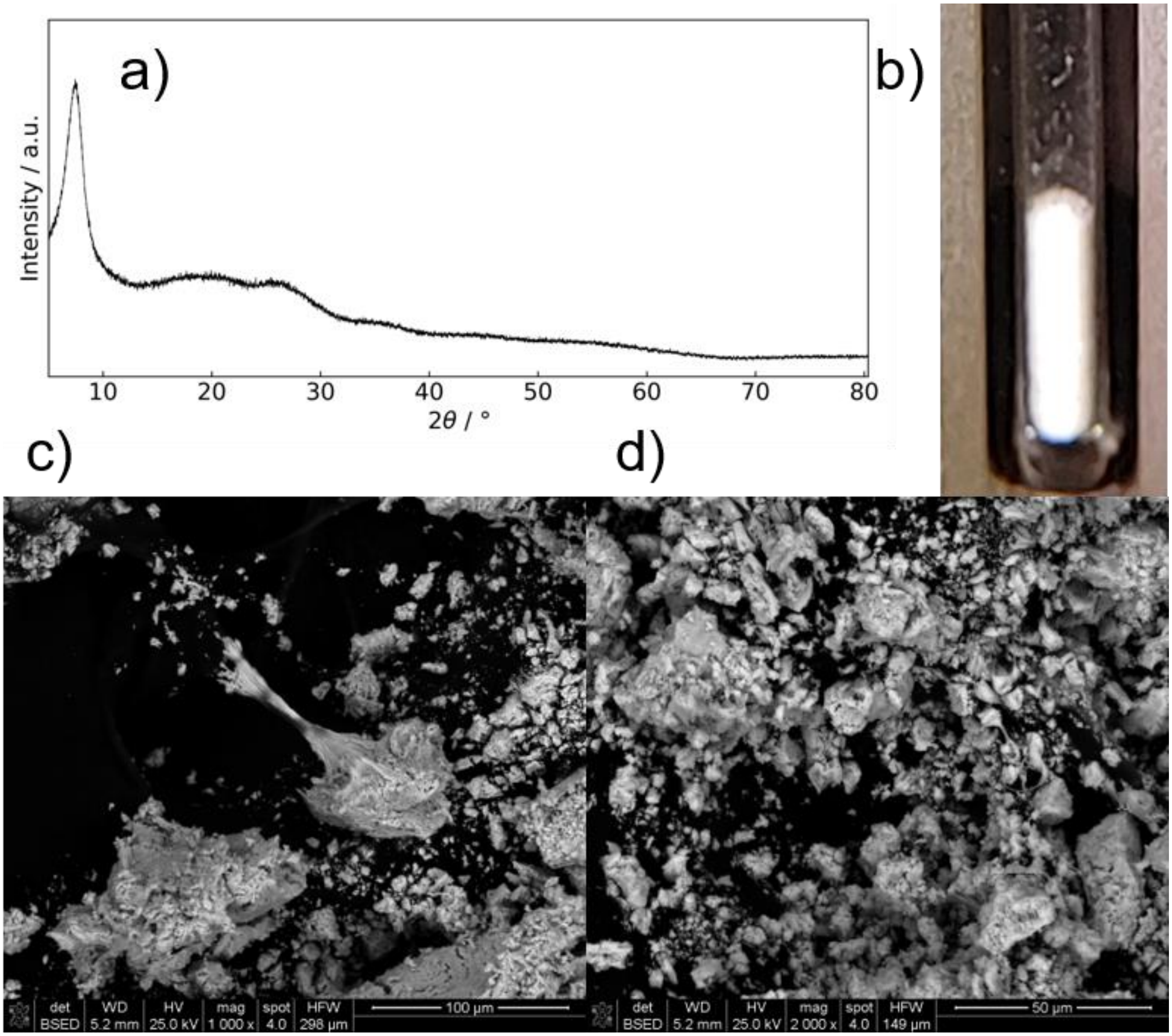


**Supplementary Figure 1.** Characterization of $Bi(TFA)_3$ precursor. Powder XRD pattern (a), photograph (b), and SEM images (c, d) of as-synthesized $Bi(TFA)_3$ powder.

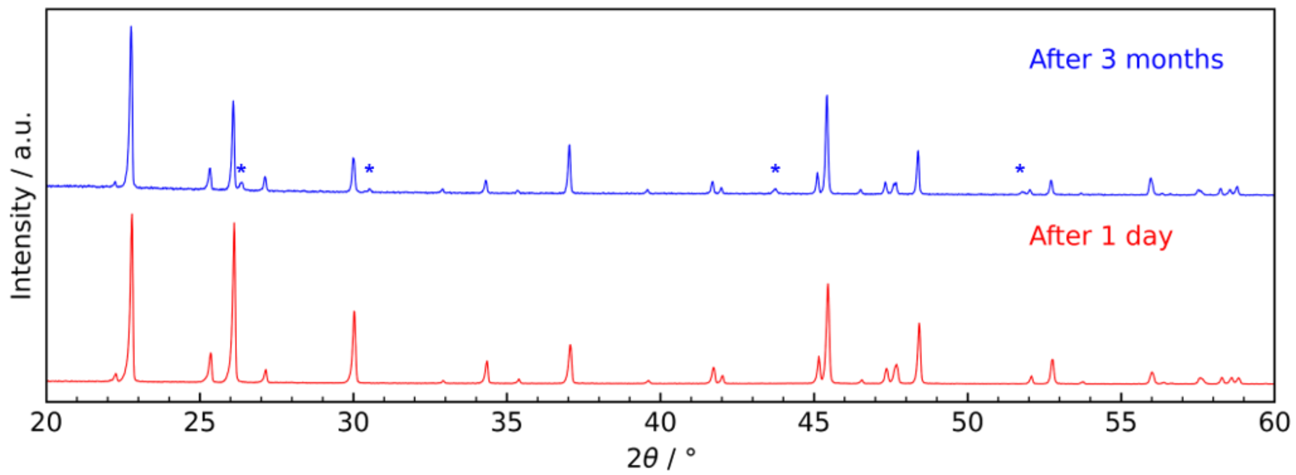


**Supplementary Figure 2**. XRD characterization of o-$BiF_3$. Powder XRD pattern of the o-$BiF_3$ product right after synthesis (red) and after 3 months under air (blue).

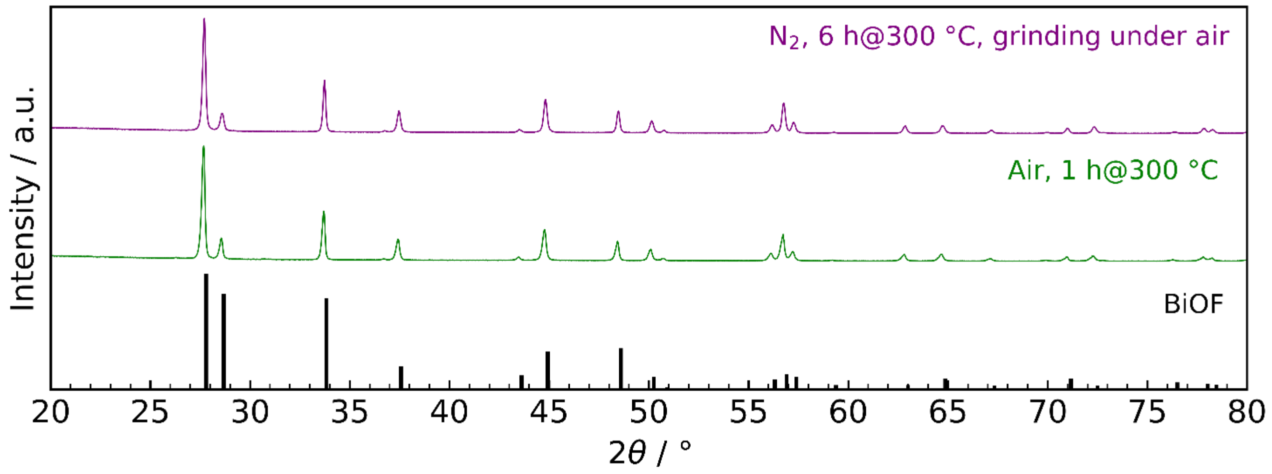


**Supplementary Figure 3.** XRD characterization of BiOF. Powder XRD pattern of BiOF synthesized from the $Bi(TFA)_3$ precursor (when heat-treated under air).

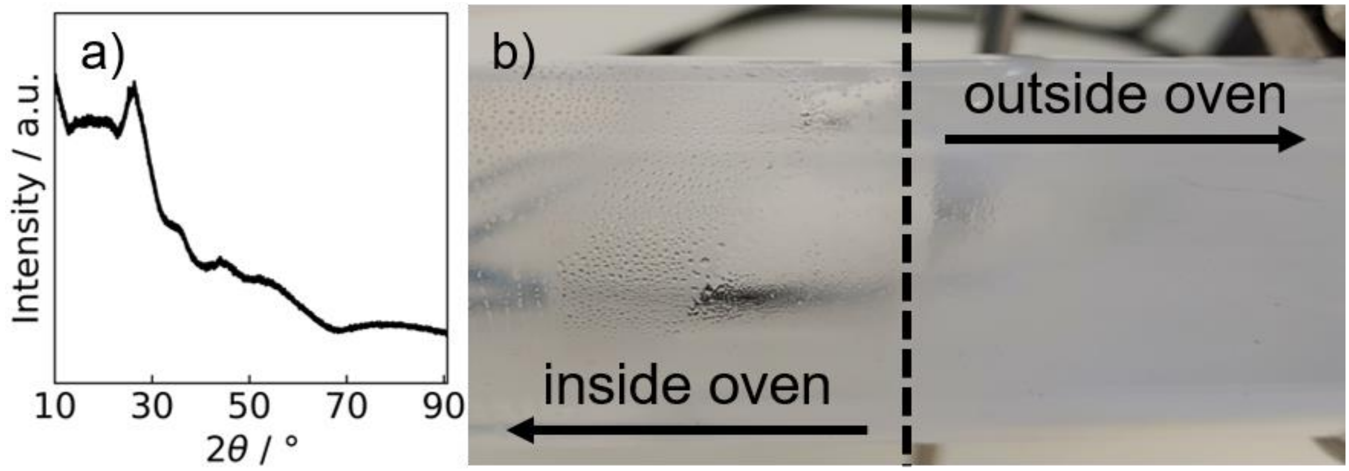


**Supplementary Figure 4.** Characterization of sublimation product of thermal decomposition of $Bi(TFA)_3$. Powder XRD pattern (a) and photograph (b) of white sublimation product outside the oven after thermal decomposition of $Bi(TFA)_3$.

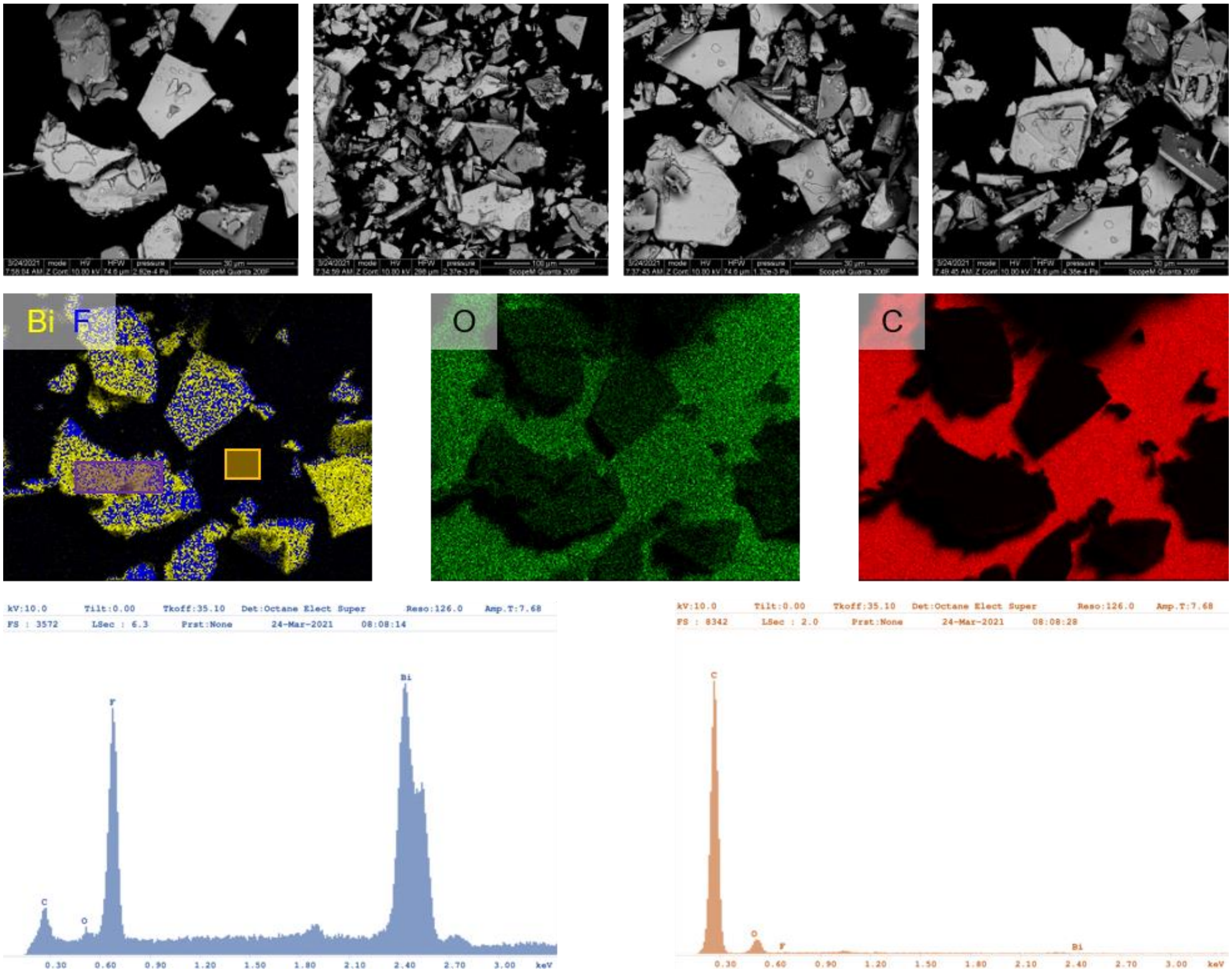


**Supplementary Figure 5**. SEM/EDX characterization of as-synthesized $BiF_3$. Representative SEM images of the as-synthesized $BiF_3$ including EDX mapping of Bi, F, O, and C and EDX spectra of the $BiF_3$ particles (blue) and the reference background (orange).

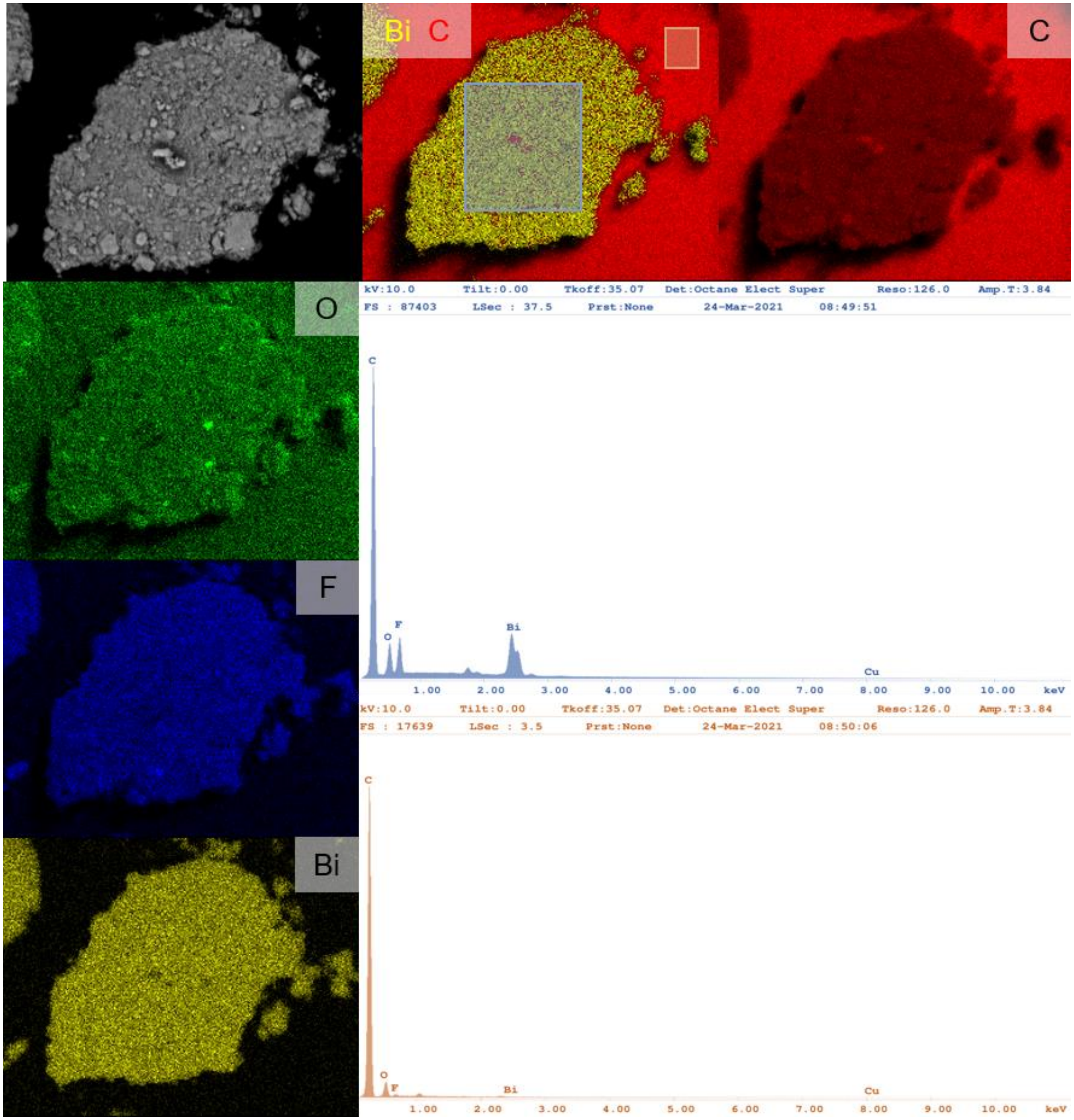


**Supplementary Figure 6.** SEM/EDX characterization of ball-milled $BiF_3$. Representative SEM images of the $BiF_3$ after ball-milling, including EDX mapping of Bi, F, O and C and EDX spectra of $BiF_3$ particles (blue) and the reference background (orange).

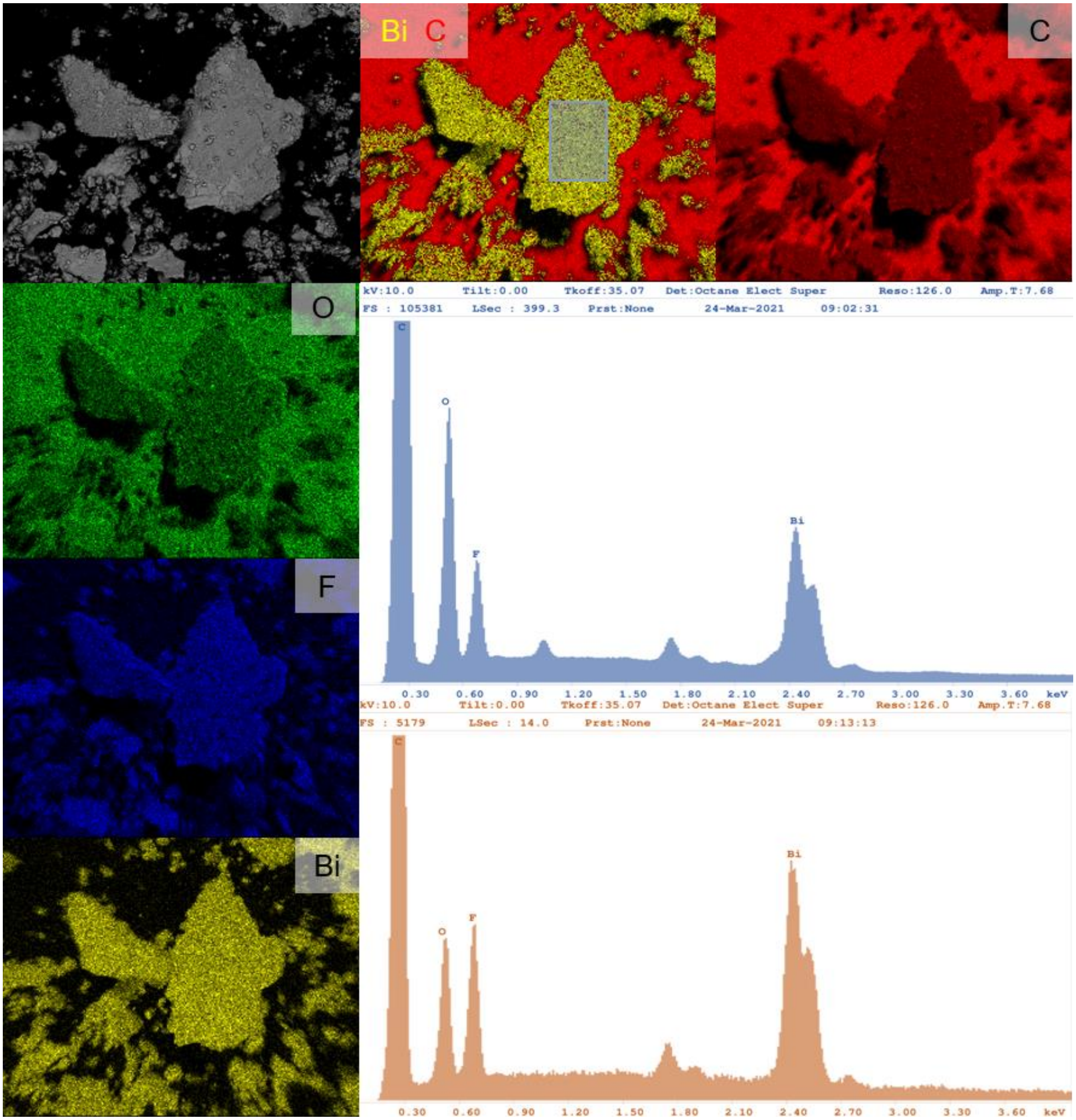


**Supplementary Figure 7.** SEM/EDX characterization of ball-milled $BiF_3$. Representative SEM images of the $BiF_3$ after ball-milling, including EDX mapping of Bi, F, O and C and EDX spectra of $BiF_3$ particles (blue) and the reference background (orange).

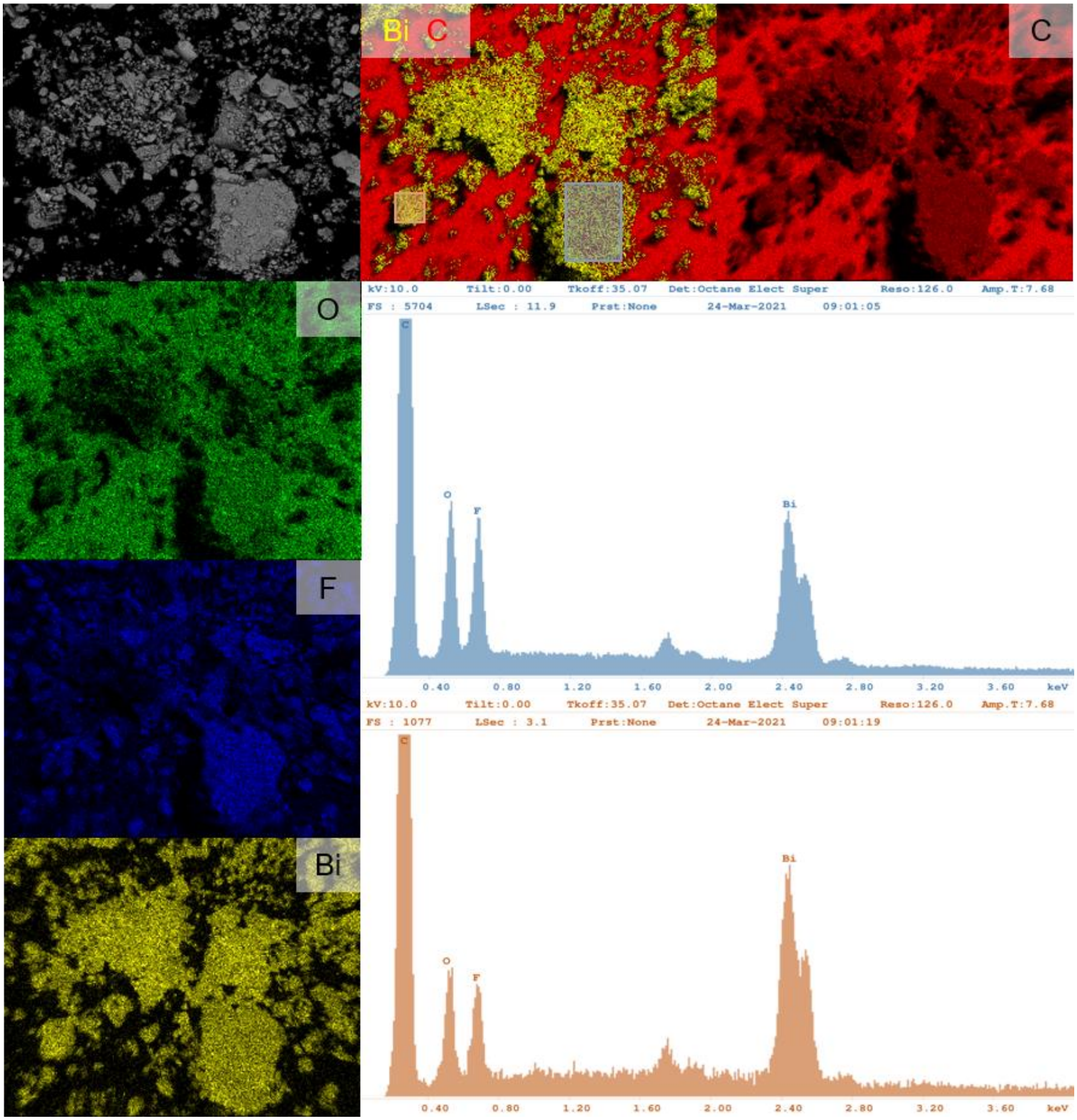


**Supplementary Figure 8.** SEM/EDX characterization of ball-milled $BiF_3$. Representative SEM images of the $BiF_3$ after ball-milling, including EDX mapping of Bi, F, O, and C and EDX spectra of $BiF_3$ particles (blue) and the reference background (orange).

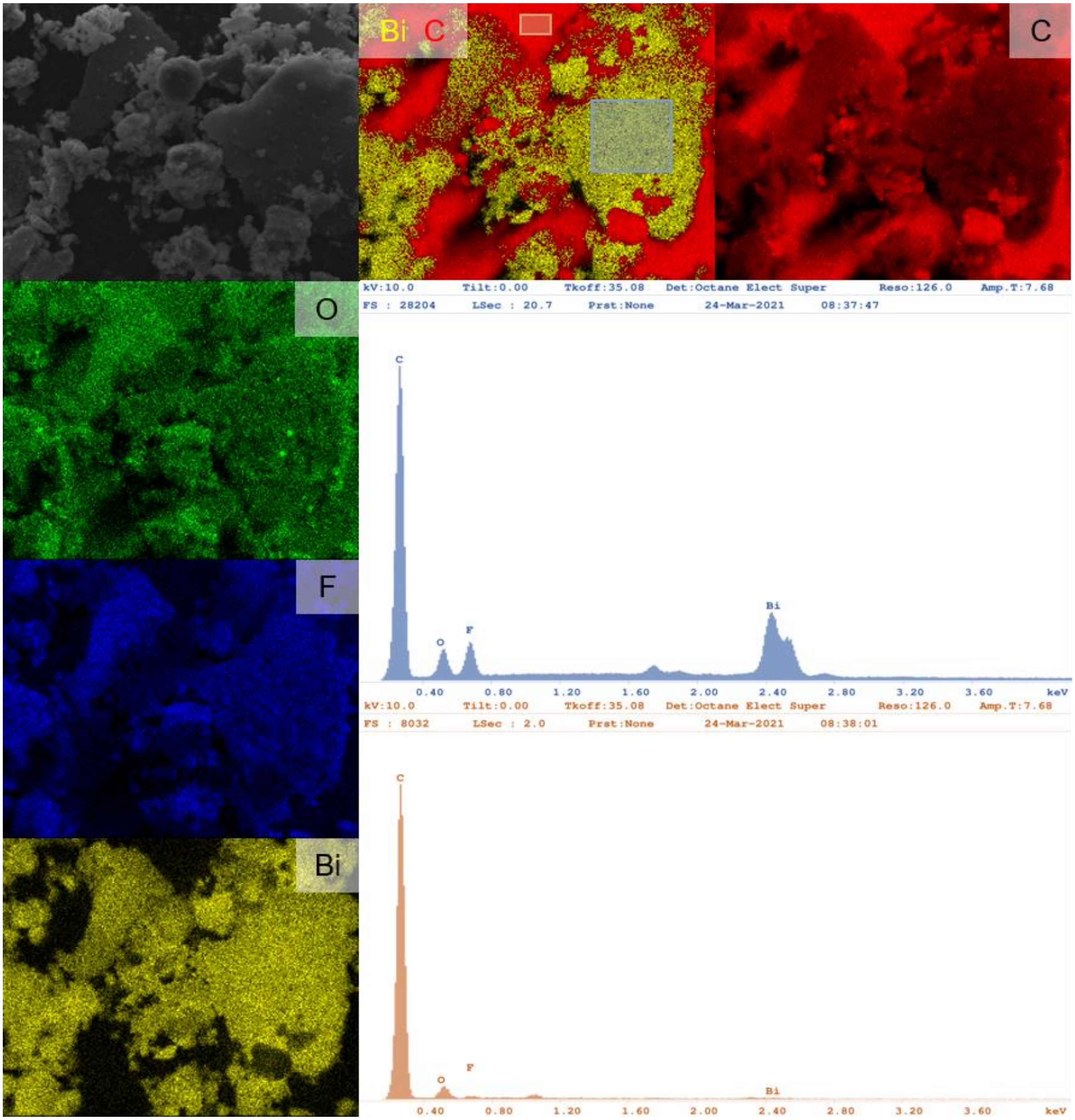


**Supplementary Figure 9.** SEM/EDX characterization of ball-milled $BiF_3$. Representative SEM images of the $BiF_3$ after ball-milling, including EDX mapping of Bi, F, O, and C and EDX spectra of $BiF_3$ particles (blue) and the reference background (orange).

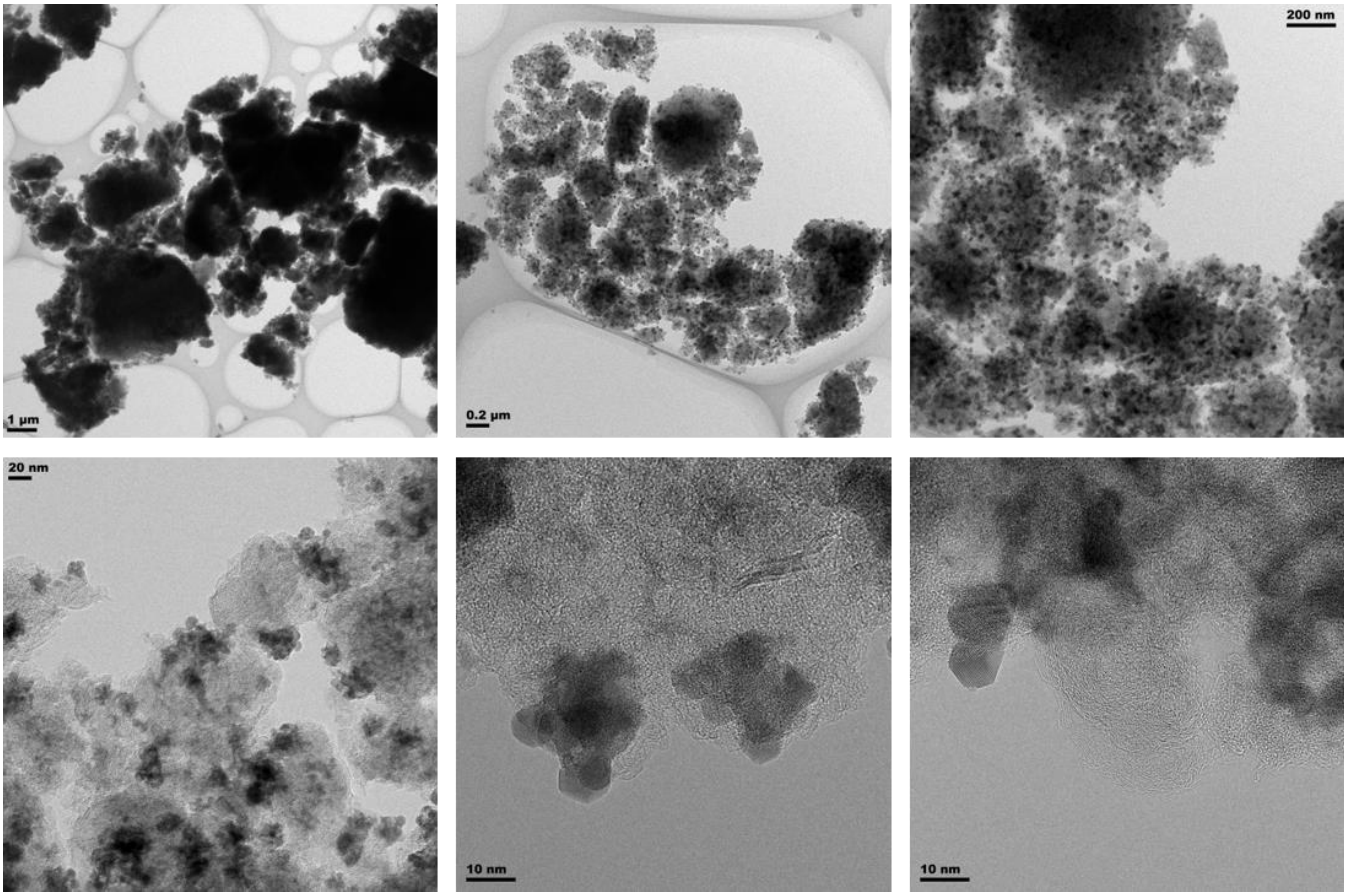


**Supplementary Figure 10.** TEM characterization of ball-milled $BiF_3$. TEM images of $BiF_3$ after ball-milling with carbon black at different magnifications.

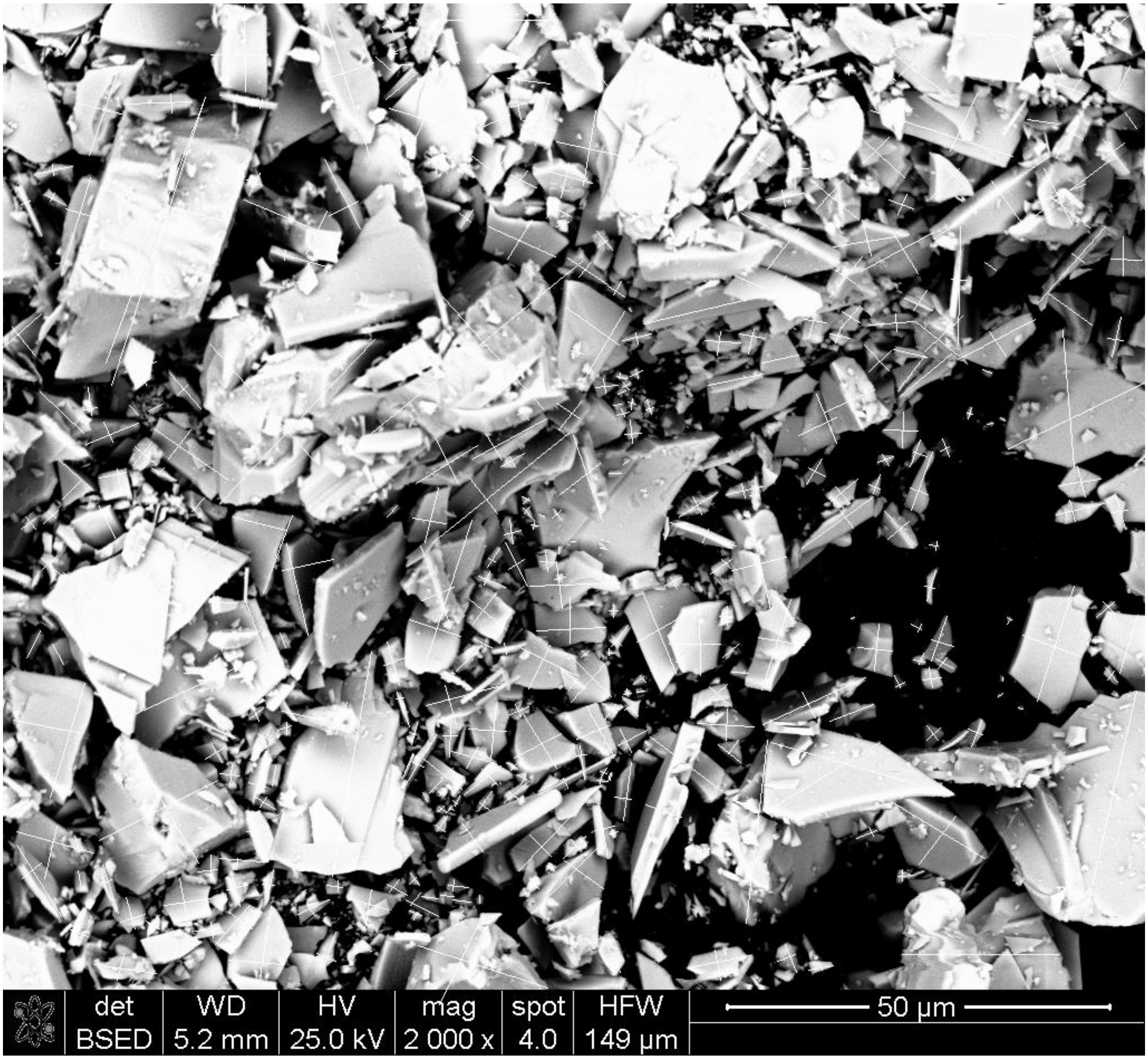


**Supplementary Figure 11.** SEM characterization of o-$BiF_3$. SEM image of the pristine o-$BiF_3$ powder before ball-milling. The white lines indicate the distances used to evaluate the particle diameters.

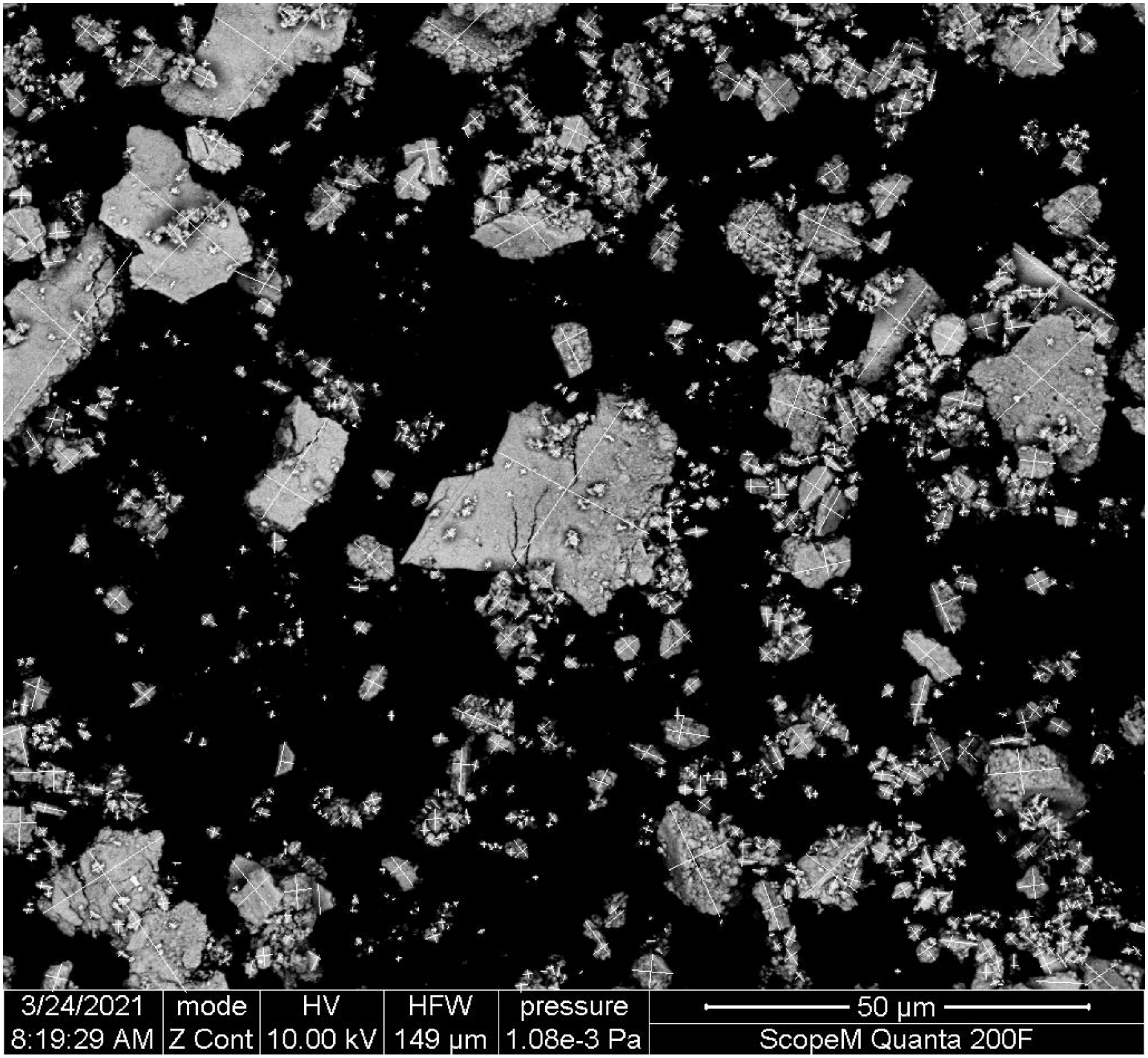


**Supplementary Figure 12.** SEM characterization of α-$BiF_3$. SEM image of the final α-$BiF_3$ powder after ball-milling. The white lines indicate the distances used to evaluate the particle diameters.

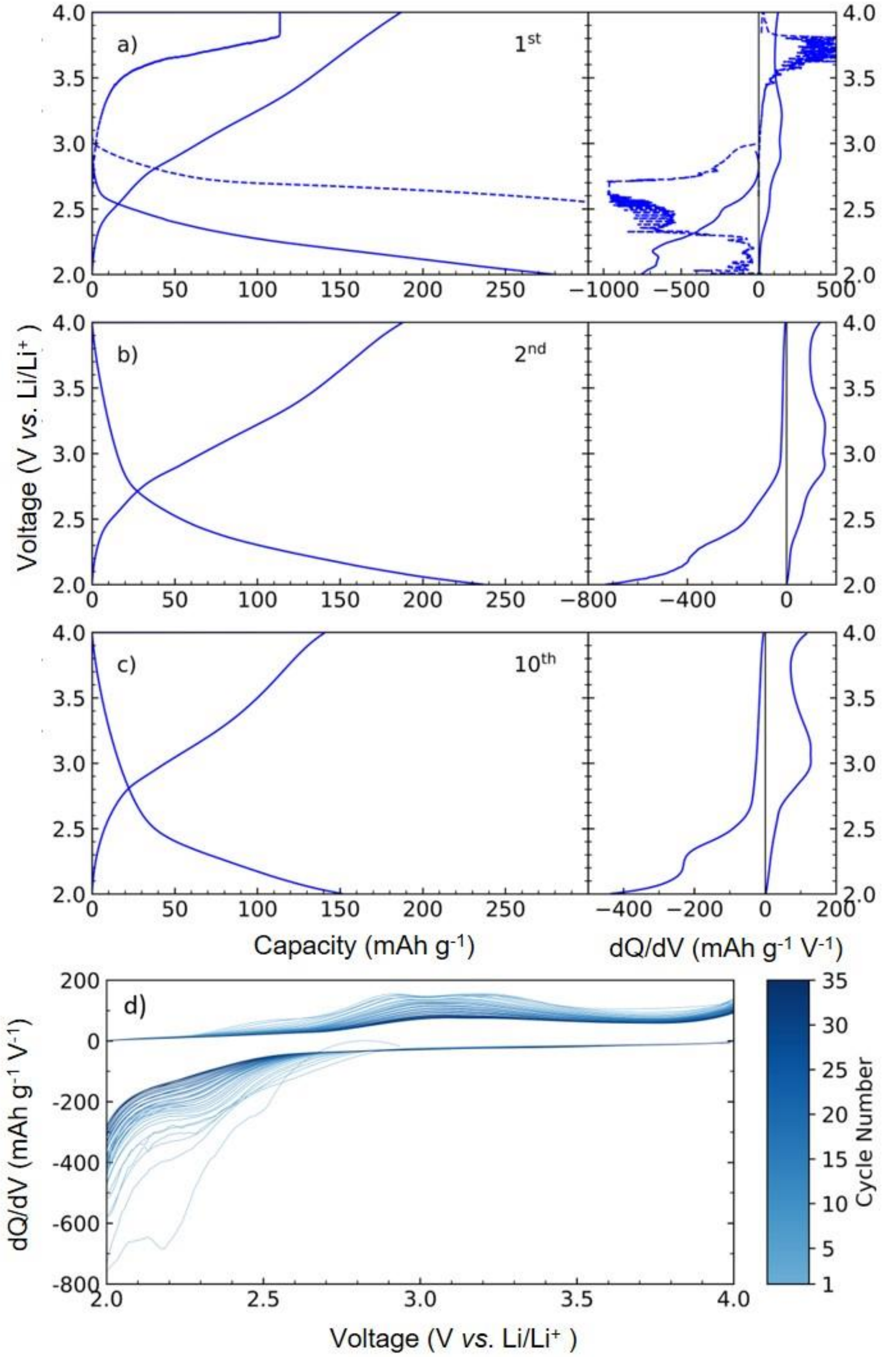


**Supplementary Figure 13.** Electrochemical performance of $BiF_3$. Charge and discharge voltage profiles and associated *dQ/dV* curves of $BiF_3$ cathode measured in $LiPF_6$ (1M and 4.3M)-EC/DMC Li-ion electrolytes for the first (a), second (b) and tenth (c) cycles. (d) Combined d*Q*/d*V* plots measured upon cycling of $BiF_3$ cathode in 1M $LiPF_6$-EC/DMC Li-ion electrolyte for first 35 cycles. The $BiF_3$/Li half cells were cycled at current density of 30 mA $g^{-1}$ in the voltage range of 2–4 V *vs.* $Li^+$/Li.

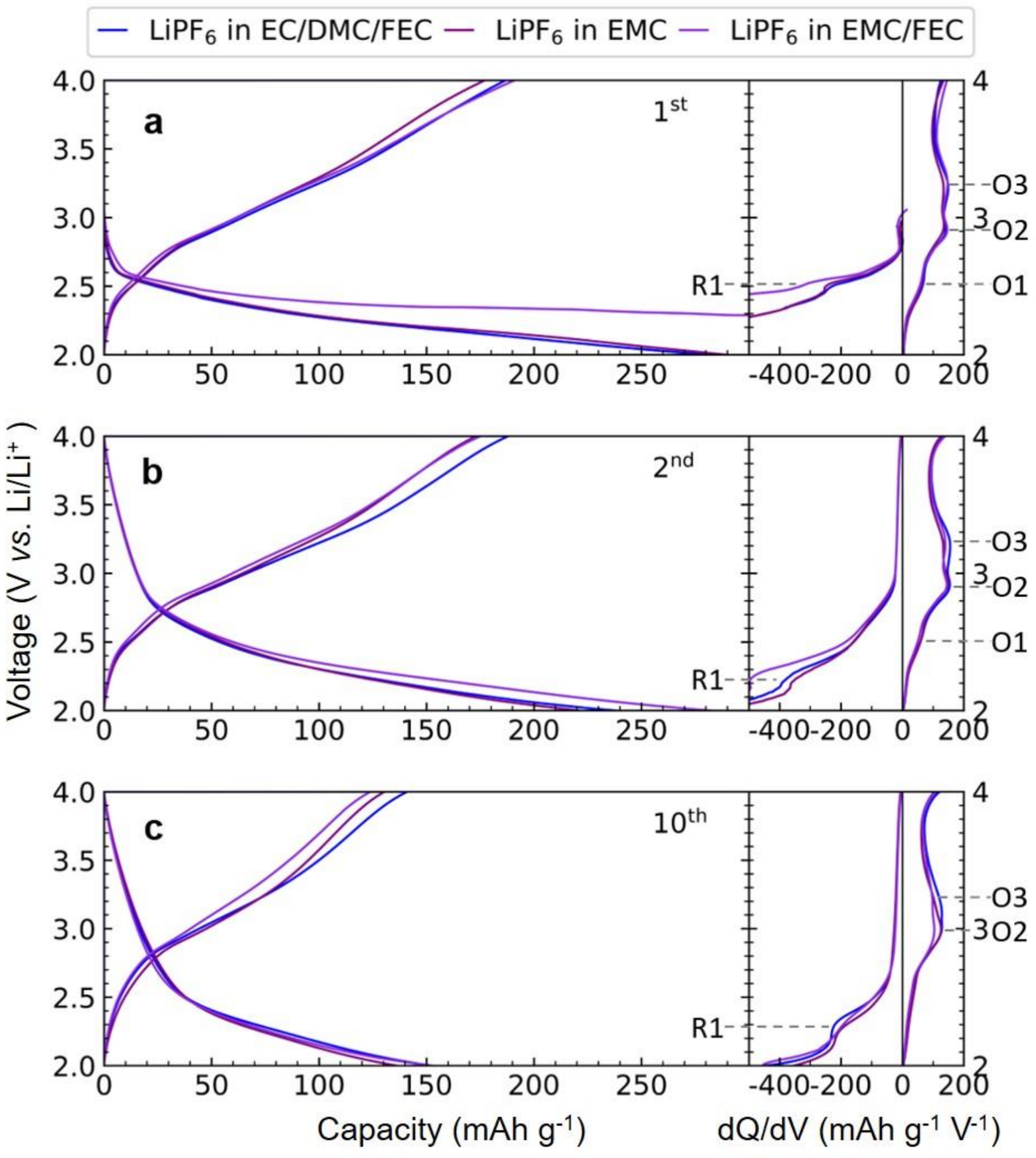


**Supplementary Figure 14.** Electrochemical performance of $BiF_3$. Charge and discharge voltage profiles and associated *dQ/dV* curves of $BiF_3$ cathode measured with 1M $LiPF_6$ in EC/DMC + 3 wt-% FEC, EMC and EMC + 3 wt-% FEC electrolytes for the first (a), second (b) and tenth (c) cycles. The $BiF_3$/Li half cells were cycled at current density of 30 mA $g^{-1}$ in the voltage range of 2–4 V *vs*. $Li^+$/Li.

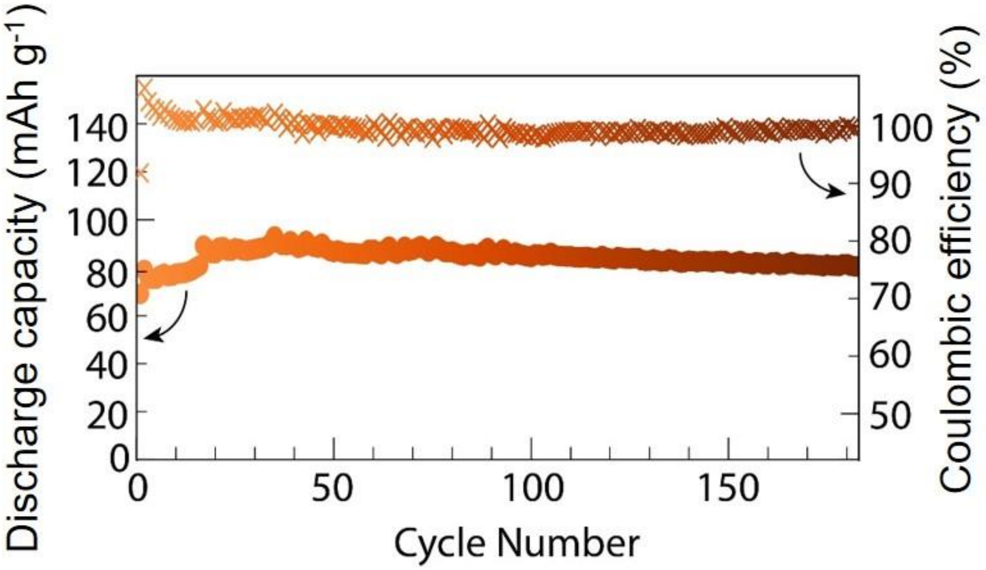


**Supplementary Figure 15.** Cyclic stability measurements of $BiF_3$. Cycling stability of $BiF_3$ cathode measured in 1M LiFSI-Pyr1,4TFSI electrolyte at current density of 30 mA $g^{-1}$ within voltage range of (2–4 V *vs*. $Li^+$/Li).

**Supplementary Table 1.** Fitted probability densities for the particle size distribution of the pristine o-$BiF_3$ powder before ball-milling. α, β, γ, μ, σ and λ are the parameters used to fit the probability distributions. The log-likelihood, the Akaike Information Criterion (AIC), the Bayesian Information Criterion (BIC) and the Anderson-Darling (AD) statistic were used to determine the goodness of the fit.

| Distribution | α | β | γ | μ | σ | λ | Log-likelihood | AICc | BIC | AD |
|---|---|---|---|---|---|---|---|---|---|---|
| Lognormal 2P | | | | 1.231 | 0.840 | | -1681 | 3366 | 3375 | 0.804 |
| Lognormal 3P | | | 0.148 | 1.167 | 0.892 | | -1678 | 3363 | 3377 | 0.425 |
| Loglogistic 3P | | | 0.235 | 1.128 | 0.533 | | -1685 | 3377 | 3391 | 1.017 |
| Loglogistic 2P | | | | 1.216 | 0.485 | | -1691 | 3386 | 3395 | 1.561 |
| Gamma 3P | 3.597 | 1.298 | 0.260 | | | | -1712 | 3430 | 3444 | 5.964 |
| Weibull 3P | 4.844 | 1.091 | 0.262 | | | | -1720 | 3447 | 3460 | 7.045 |
| Exponential 2P | | | 0.263 | | | 0.214 | -1725 | 3454 | 3463 | 9.322 |
| Gamma 2P | 3.247 | 1.518 | | | | | -1730 | 3465 | 3474 | 8.376 |
| Weibull 2P | 5.247 | 1.170 | | | | | -1747 | 3499 | 3508 | 10.434 |
| Exponential 1P | | | | | | 0.203 | -1762 | 3526 | 3531 | 16.933 |
| Normal 2P | | | | 4.930 | 5.018 | | -2059 | 4122 | 4131 | 50.950 |
| Gumbel 2P | | | | 7.967 | 8.700 | | -2385 | 4774 | 4783 | |

The lognormal 2P distribution was determined to have the best fit based on the BIC. The distribution is characterized by the scale parameter $\mu = 1.23 \pm 0.03$ and a shape parameter $\sigma = 0.84 \pm 0.02$.

**Supplementary Table 2.** Fitted probability densities for the particle size distribution of the final α-$BiF_3$ powder after ball-milling. α, β, γ, μ, σ and λ are the parameters used to fit the probability distributions. The log-likelihood, the Akaike Information Criterion (AIC), the Bayesian Information Criterion (BIC) and the Anderson-Darling (AD) statistic were used to determine the goodness of the fit.

| Distribution | α | β | γ | μ | σ | λ | Log-likelihood | AICc | BIC | AD |
|---|---|---|---|---|---|---|---|---|---|---|
| Loglogistic 3P | | | 0.060 | -0.019 | 0.437 | | -2639 | 5284 | 5301 | 3.201 |
| Loglogistic 2P | | | | 0.047 | 0.408 | | -2651 | 5307 | 5318 | 5.146 |
| Lognormal 3P | | | 0.036 | 0.039 | 0.776 | | -2687 | 5380 | 5397 | 9.684 |
| Lognormal 2P | | | | 0.084 | 0.744 | | -2693 | 5390 | 5401 | 11.298 |
| Gamma 3P | 0.991 | 1.459 | 0.071 | | | | -2967 | 5940 | 5957 | 55.589 |
| Gamma 2P | 0.922 | 1.646 | | | | | -3018 | 6039 | 6051 | 63.059 |
| Weibull 3P | 1.502 | 1.085 | 0.073 | | | | -3036 | 6079 | 6096 | 73.397 |
| Exponential 2P | | | 0.073 | | | 0.692 | -3052 | 6108 | 6119 | 88.328 |
| Weibull 2P | 1.607 | 1.135 | | | | | -3123 | 6251 | 6262 | 86.608 |
| Exponential 1P | | | | | | 0.659 | -3162 | 6326 | 6331 | 114.671 |
| Normal_2P | | | | 1.518 | 1.942 | | -4646 | 9296 | 9308 | |
| Gumbel_2P | | | | 2.876 | 5.097 | | -6459 | 12922 | 12934 | |

The loglogistic 3P distribution was determined to have the best fit based on the BIC. Nevertheless, the loglogistic 2P distribution was chosen to reduce the complexity of the model fit and because it has almost identical goodness of fit. The distribution is characterized by the scale parameter $\mu = 0.0474 \pm 0.007$ and a shape parameter $\sigma = 0.408 \pm 0.007$.

**Supplementary Table 3.** Comparison of the electrochemical performance of $BiF_3$ (present work) with other reported systems comprising metal fluorides and alkali metal fluorides as cathode materials for lithium-ion batteries.

| Cathode material | Current density ($mAh\ g^{-1}$ or $mAh\ cm^{-2}$) | Initial capacity ($mAhg^{-1}$) | Retained capacity ($mAhg^{-1}$) | Cycle number | Potential range (V vs. $Li^+/Li$) |
|---|---|---|---|---|---|
| $BiF_3$ (present work) | 30 mA $g^{-1}$ | 208 | 96 | 80 | 2-4 |
| Tysonite-$BiF_3$[1] | 45.45 mA $g^{-1}$ | 235 | 190 | 15 | 2-4.5 |
| Orthorhombic -$BiF_3$[1] | 45.45 mA $g^{-1}$ | 220 | 185 | 15 | 2-4.5 |
| Tysonite-$BiF_3$[2] | 40 mA $g^{-1}$ | 200 | 120 | 20 | 2-4 |
| Orthorhombic -$BiF_3$[3] | 15 mA $g^{-1}$ | 250 | 220 | 50 | 2-4.5 |
| Orthorhombic -$BiF_3$[4] | 15 mA $g^{-1}$ | 250 | 183 | 100 | 0.55 V – 1.95 V vs. $LiCoO_2$ |
| $BiF_3$ nanocrystals[5] | 50 mAh $g^{-1}$ | 320 | 170 | 10 | 2.0-4.0 |
| $FeF_2$ nanorods[6] | 200 mAh $g^{-1}$ | 500 | 100 | 30 | 1.5-4 |
| $FeF_3$ nanorods[6] | 50 mAh $g^{-1}$ | 125 | 110 | 35 | 2.3-4 |
| $FeF_2$ nanorods[7] | 28.5 mA $g^{-1}$ | 700 | 525 | 50 | 1.2-4.0 |
| | | | | | |
| $NaFeF_3$ nanoplates[8] | 200 mAh $g^{-1}$ | 183 | 95 | 200 | 2.0-4.5 |
| $FeF_3$[9] | 0.2 mAh $cm^{-2}$ | 140 | 80 | 2 | 2.0-4.5 |
| $TiF_3$[9] | 0.2 mAh $cm^{-2}$ | 180 | 80 | 2 | 2.0-4.5 |
| $FeF_3$:C nanocomposite[10] | 7.58 mAh $g^{-1}$ T=70°C | 700 | 600 | 12 | 1.5-4.5 |
| $Na_3FeF_6$[11] | 50 mAh $g^{-1}$ | 428 | 296.7 | 60 | 1.0-4.5 |
| $FeF_3$-C composite[12] | 0.2 mAh $cm^{-2}$ | 200 | 160 | 40 | 2.0-4.5 |

| | | | | | |
|---|---|---|---|---|---|
| Nano flake/micro-flower $FeF_3$[13] | 237 mAh $g^{-1}$ | 187.1 | 172.3 | 50 | 2.0-4.5 |
| $FeF_3$ NPs[14] | 23.7-237 mAh $g^{-1}$ | 270 | 150 | 55 | 1.5-4.5 |
| Nanocrystalline $FeF_3$[15] | 100 mAh $g^{-1}$ | 220 | 150 | 100 | 2.0-4.2 |
| $FeF_3$ nanoflowers on CNT[16] | 20 mAh $g^{-1}$ | 210 | 200 | 30 | 2.0-4.5 |
| Iron Fluoride@CMK-3 Nanocomposite[17] | 23.7-2370 mAh $g^{-1}$ | 200 | 180 | 45 | 1.5-4.5 |
| Iron Fluoride@CMK-3 Nanocomposite[17] | 2.37- 11.85 Ah $g^{-1}$ | 110 | 105 | 145 | 1.5-4.5 |
| $FeF_3{*}0.33H_2O$[18] | 71 mAh $g^{-1}$ | 126 | 105 | 30 | 1.6-4.5 |
| $FeF_3$ nanowires [19] | 50 mAh $g^{-1}$ | 543 | 223 | 50 | 1.5-4.5 |
| $FeF_3$ - graphene nanocomposites[20] | 45 mAh $g^{-1}$ | 210 | 205 | 30 | 2.0-4.5 |
| $FeF_3$ - graphene nanocomposites[20] | 71.2 mAh $g^{-1}$ | 620 | 490 | 20 | 1.5-4.5 |
| Macroporous $FeF_3$[21] | 20 mAh $g^{-1}$ | 210 | 190 | 30 | 2.0-4.5 |
| Graphene-wrapped $FeF_3$ nanocrystals[22] | 20.8 mAh $g^{-1}$ | 280 | 185.6 | 100 | 1.5-4.5 |
| $FeF_3$[23] | 20 mAh $g^{-1}$ | 224 | 143 | 100 | 2.0-4.5 |
| $FeF_3{*}0.33H_2O$/C nanocomposites[24] | 119 mAh $g^{-1}$ | 160 | 140 | 100 | 2.0-4.5 |
| $FeF_3$ nanocomposite[25] | 10-80 mAh $g^{-1}$ | 200 | 175 | 55 | 2.0-4.5 |

## Supplementary References


1. Bervas, M; Badway, F; Klein, LC; Amatucci, GG, Bismuth fluoride nanocomposite as a positive electrode material for rechargeable lithium batteries. *Electrochem. Solid-State Lett.* **2005,** 8, A179.
2. Bervas, M; Mansour, AN; Yoon, WS; Al-Sharab, JF; Badway, F; Cosandey, F; Klein, LC; Amatucci, GG, Investigation of the lithiation and delithiation conversion mechanisms of bismuth fluoride nanocomposites. *J. Electrochem. Soc.* **2006,** 153, A799.
3. Gmitter, AJ; Badway, F; Rangan, S; Bartynski, RA; Halajko, A; Pereira, N; Amatucci, GG, Formation, dynamics, and implication of solid electrolyte interphase in high voltage reversible conversion fluoride nanocomposites. *J. Mater. Chem.* **2010,** 20, 4149-4161.
4. Gmitter, AJ; Gural, J; Amatucci, GG, Electrolyte development for improved cycling performance of bismuth fluoride nanocomposite positive electrodes. *J. Power Sources* **2012,** 217, 21-28.
5. Oszajca, MF; Kravchyk, KV; Walter, M; Krieg, F; Bodnarchuk, MI; Kovalenko, MV, Colloidal $BiF_3$ nanocrystals: A bottom-up approach to conversion-type Li-ion cathodes. *Nanoscale* **2015,** 7, 16601-16605.
6. Guntlin, CP; Kravchyk, KV; Erni, R; Kovalenko, MV, Transition metal trifluoroacetates (M = Fe, Co, Mn) as precursors for uniform colloidal metal difluoride and phosphide nanoparticles. *Sci. Rep.* **2019,** 9, 6613.
7. Xiao, AW; Lee, HJ; Capone, I; Robertson, A; Wi, T-U; Fawdon, J; Wheeler, S; Lee, H-W; Grobert, N; Pasta, M, Understanding the conversion mechanism and performance of monodisperse $FeF_2$ nanocrystal cathodes. *Nat. Mater.* **2020,** 19, 644-654.
8. Kravchyk, KV; Zünd, T; Wörle, M; Kovalenko, MV; Bodnarchuk, MI, $NaFeF_3$ nanoplates as low-cost sodium and lithium cathode materials for stationary energy storage. *Chem. Mater.* **2018,** 30, 1825-1829.
9. Arai, H; Okada, S; Sakurai, Y; Yamaki, J-i, Cathode performance and voltage estimation of metal trihalides. *J. Power Sources* **1997,** 68, 716-719.
10. Badway, F; Cosandey, F; Pereira, N; Amatucci, GG, Carbon metal fluoride nanocomposites: high-capacity reversible metal fluoride conversion materials as rechargeable positive electrodes for Li batteries. *J. Electrochem. Soc.* **2003,** 150, A1318-A1327.
11. Sun, S; Shi, Y; Bian, S; Zhuang, Q; Liu, M; Cui, Y, Enhanced charge storage of $Na_3FeF_6$ with carbon nanotubes for lithium-ion batteries. *Solid State Ionics* **2017,** 312, 61-66.
12. Nishijima, M; Gocheva, ID; Okada, S; Doi, T; Yamaki, J-i; Nishida, T, Cathode properties of metal trifluorides in Li and Na secondary batteries. *J. Power Sources* **2009,** 190, 558-562.
13. Bai, Y; Zhou, X; Zhan, C; Ma, L; Yuan, Y; Wu, C; Chen, M; Chen, G; Ni, Q; Wu, F; Shahbazian-Yassar, R; Wu, T; Lu, J; Amine, K, 3D Hierarchical nano-flake/micro-flower iron fluoride with hydration water induced tunnels for secondary lithium battery cathodes. *Nano Energy* **2017,** 32, 10-18.
14. Di Carlo, L; Conte, DE; Kemnitz, E; Pinna, N, Microwave-assisted fluorolytic sol-gel route to iron fluoride nanoparticles for Li-ion batteries. *Chem. Commun.* **2014,** 50, 460-462.
15. Guntlin, CP; Zünd, T; Kravchyk, KV; Wörle, M; Bodnarchuk, MI; Kovalenko, MV, Nanocrystalline $FeF_3$ and $MF_2$ (M = Fe, Co, and Mn) from Metal Trifluoroacetates and Their Li(Na)-Ion Storage Properties. *J. Mater. Chem. A* **2017,** 5, 7383-7393.

16. Kim, SW; Seo, DH; Gwon, H; Kim, J; Kang, K, Fabrication of $FeF_3$ nanoflowers on CNT branches and their application to high power lithium rechargeable batteries. *Advanced materials* **2010,** 22, 5260-5264.
17. Li, B; Zhang, N; Sun, K, Confined iron fluoride@CMK-3 nanocomposite as an ultrahigh rate capability cathode for Li-ion batteries. *Small* **2014,** 10, 2039-2046.
18. Li, C; Gu, L; Tsukimoto, S; van Aken, PA; Maier, J, Low-temperature ionic-liquid-based synthesis of nanostructured iron-based fluoride cathodes for lithium batteries. *Advanced materials* **2010,** 22, 3650-3654.
19. Li, L; Meng, F; Jin, S, High-capacity lithium-ion battery conversion cathodes based on iron fluoride nanowires and insights into the conversion mechanism. *Nano Lett.* **2012,** 12, 6030-6037.
20. Liu, J; Wan, Y; Liu, W; Ma, Z; Ji, S; Wang, J; Zhou, Y; Hodgson, P; Li, Y, Mild and cost-effective synthesis of iron fluoride-graphene nanocomposites for high-rate Li-ion battery cathodes. *J. Mater. Chem. A* **2013,** 1, 1969-1975.
21. Ma, DL; Cao, ZY; Wang, HG; Huang, XL; Wang, LM; Zhang, XB, Three-dimensionally ordered macroporous $FeF_3$ and its in situ homogenous polymerization coating for high energy and power density lithium ion batteries. *Energy Environ. Sci.* **2012,** 5, 8538-8542.
22. Ma, R; Lu, Z; Wang, C; Wang, HE; Yang, S; Xi, L; Chung, JC, Large-scale fabrication of graphene-wrapped $FeF_3$ nanocrystals as cathode materials for lithium ion batteries. *Nanoscale* **2013,** 5, 6338-6343.
23. Myung, ST; Sakurada, S; Yashiro, H; Sun, YK, Iron trifluoride synthesized via evaporation method and its application to rechargeable lithium batteries. *J. Power Sources* **2013,** 223, 1-8.
24. Tan, JL; Liu, L; Hu, H; Yang, ZH; Guo, HP; Wei, QL; Yi, X; Yan, ZC; Zhou, Q; Huang, ZF; Shu, HB; Yang, XK; Wang, XY, Iron fluoride with excellent cycle performance synthesized by solvothermal method as cathodes for lithium ion batteries. *J. Power Sources* **2014,** 251, 75-84.
25. Yabuuchi, N; Sugano, M; Yamakawa, Y; Nakai, I; Sakamoto, K; Muramatsu, H; Komaba, S, Effect of heat-treatment process on $FeF_3$ nanocomposite electrodes for rechargeable Li batteries. *J. Mater. Chem.* **2011,** 21, 10035-10041.
